# Exploring the Genetic Diversity and Molecular Evolution of Seoul and Hantaan Orthohantaviruses

**DOI:** 10.3390/v16010105

**Published:** 2024-01-11

**Authors:** Atanas V. Demirev, Sangyi Lee, Sejik Park, Hyunbeen Kim, Seunghye Cho, Kyuyoung Lee, Kisoon Kim, Jin-Won Song, Man-Seong Park, Jin Il Kim

**Affiliations:** 1 Department of Microbiology, Institute for Viral Diseases, Korea University College of Medicine, Seoul 02841, Republic of Koreasylee721@korea.ac.kr (S.L.); psj0817@korea.ac.kr (S.P.); kimhb225@korea.ac.kr (H.K.); shcho7003@korea.ac.kr (S.C.); wing290@korea.ac.kr (K.L.); tigerkis@korea.ac.kr (K.K.); jwsong@korea.ac.kr (J.-W.S.); 2 Vaccine Innovation Center, Korea University College of Medicine, Seoul 02841, Republic of Korea; 3 Biosafety Center, Korea University College of Medicine, Seoul 02841, Republic of Korea

**Keywords:** evolution, Hantaan virus, phylogeny, reassortment, Seoul virus

## Abstract

Seoul (SEOV) and Hantaan (HTNV) orthohantaviruses are significant zoonotic pathogens responsible for hemorrhagic fever with renal syndrome. Here, we investigated the molecular evolution of SEOV and HTNV through phylogenetic and bioinformatic analyses using complete genome sequences of their large (L), medium (M), and small (S) gene segments. Despite similar epizootic cycles and clinical symptoms, SEOV and HTNV exhibited distinct genetic and evolutionary dynamics. The phylogenetic trees of each segment consistently showed major genetic clades associated with the geographical distribution of both viruses. Remarkably, SEOV M and S segments exhibit higher evolutionary rates, rapidly increasing genetic diversity, and a more recent origin in contrast to HTNV. Reassortment events were infrequent, but both viruses appear to utilize the M gene segment in genetic exchanges. SEOV favors the L or M segment reassortment, while HTNV prefers the M or S segment exchange. Purifying selection dominates in all three gene segments of both viruses, yet SEOV experiences an elevated positive selection in its glycoprotein Gc ectodomain. Key amino acid differences, including a positive ‘lysine fence’ (through residues K77, K82, K231, K307, and K310) located at the tip of the Gn, alongside the physical stability around an RGD-like motif through M108-F334 interaction, may contribute to the unique antigenic properties of SEOV. With the increasing global dispersion and potential implications of SEOV for the global public health landscape, this study highlights the unique evolutionary dynamics and antigenic properties of SEOV and HTNV in informing vaccine design and public health preparedness.

## 1. Introduction

Hemorrhagic fever with renal syndrome (HFRS) is a zoonotic disease caused by rodent-borne orthohantaviruses, which frequently give rise to outbreaks primarily in Europe and Eastern Asia [1]. Among the viruses contributing to HFRS in Eurasia are Seoul (SEOV) and Hantaan (HTNV) orthohantavirus [2]. However, HTNV primarily infects *Apodemus agrarius*, and SEOV is known to infect *Rattus norvegicus* and *Rattus rattus* [2]. Recent reports show that SEOV exhibits a human case fatality rate of less than 1%, in contrast to the 1% case fatality rate associated with HTNV [3]. However, the recent detection of human infections caused by SEOV in Europe, Africa, and other regions garners increased attention towards this virus [4,5], while HTNV remains endemic in Eastern Asia [6].

Both SEOV and HTNV are enveloped RNA viruses bearing tripartite single-stranded, negative-sense RNA genome segments that are named large (L), medium (M), and small (S) genes, based on their size [7]. The L segment encodes the viral RNA-dependent RNA polymerase. The M segment codes for the envelope glycoprotein precursor that is cleaved into two glycoproteins (Gn and Gc), and the S segment codes for the nucleocapsid (N) protein. Previously, the phylogenetic analyses of the M and S segments of SEOV and HTNV have consistently revealed their distinct genetic lineages, characterized by the relatively low levels of genetic diversity within each species [8,9]. Moreover, a molecular clock analysis suggests that the most recent common ancestor of HTNV existed over 800 years ago [10]. While natural reassortment events leading to the emergence of novel genotypes have been reported in closely related HTNV variants [9], such events have not been observed in SEOV. Despite recent efforts to independently investigate the phylogenetic characteristics and genetic diversity of SEOV and HTNV [10,11], the precise mechanisms governing their emergence and distinct evolutionary dynamics remain unclear.

In light of the increasing prevalence of HFRS across Europe and its potentially significant public health implications [5,12], there is a need for a comprehensive comparative study of SEOV and other hantaviruses. This study aims to elucidate critical similarities and differences in molecular evolution between SEOV and HTNV, encompassing their genetic diversification, genome reassortment potential, glycoprotein adaptation, and their respective contributions to viral emergence, evolution, and potential impacts on public health.

## 2. Materials and Methods

### 2.1. Genome Sequence Datasets

We constructed two genomic datasets for SEOV and HTNV by compiling all available complete genome sequences of the L, M, and S segments obtained from the Bacterial and Viral Bioinformatics Resource Center (BV-BRC) complemented with the databases of the National Center for Biotechnology Information (NCBI). The gene sequences were aligned using the MAFFT software (v7.419) [13] and subsequently trimmed to ensure equal lengths for each segment, starting from the ATG codon. In the HTNV dataset, the Nc167 strain was intentionally excluded due to its classification as the Da Bie Shan virus and its apparent deviation from the M segment phylogenetic cluster of HTNV [14]. Additionally, we trimmed the S gene sequences in the SEOV dataset as we identified a truncated N-terminal in 22 out of the 80 strains. The resulting datasets comprise 80 genomes for SEOV and 146 genomes for HTNV. The genomic lengths of the L, M, and S segments are 6288 bp, 3399 bp, and 939 bp for SEOV and 6453 bp, 3405 bp, and 1287 bp for HTNV, respectively (Supplementary Materials).

### 2.2. Phylogenetic Analyses

Phylogenic relationships for each gene segment (L, M, and S) were reconstructed using the time-framed Bayesian inference method implemented using the Bayesian Evolutionary Analysis by Sampling Trees (BEAST) (v1.10.4) [15] with GTR + I + G nucleotide substitution and lognormal uncorrelated relaxed clock models. Markov Chain Monte Carlo (MCMC) chains were set to run a minimum of 400 million iterations with sampling at every 400,000 steps until all effective sample sizes exceeded 200 after a 10% burn-in. The posterior distributions of trees were summarized as the maximum clade credibility (MCC) trees using TreeAnnotator (v1.10.4) [15]. The genetic clades in SEOV and HTNV phylogenies were defined based on the identified strains in the phylogenetic trees of the M segments of the previous investigations [8,10].

### 2.3. Genetic Reassortment in SEOV and HTNV

Potential genetic reassortments among the L, M, and S segments of SEOV and HTNV were assessed using two independent methods: (1) the Markov Chain Monte Carlo (MCMC) framework for the coalescent with reassortment (CoalRe) [16] and (2) the graph-incompatibility-based reassortment finder program (GiRaF) [17]. Assuming that each segment evolved independently (CoalRe), we incorporated the reassortment rate (per lineage per year) as a parameter. To visualize the network trees in a tanglegram, we utilized a Python-based tool called baltic (https://github.com/evogytis/baltic; accessed on 20 July 2023). Reassortment events were considered reliable in the network trees if they exhibited a confidence level of greater than 0.9.

### 2.4. Genetic Diversity and Natural Selection Profiles

We employed Bayesian Skygrid plots, with data generated using Tracer (v1.7.1), to visualize the relative genetic diversity and estimate the time of the most recent common ancestors [18]. The nucleotide substitutions per site per year (dN/dS, also known as ω) were estimated from the ratio of nonsynonymous (dN) vs. synonymous (dS) using the local HyPhy package (Table 1) [19].

To identify the potential sites of positive and negative selection, we estimated natural selection profiles for all gene segments in both complete genome sets. We used three different methods, SLAC (single-likelihood ancestor counting), FUBAR (fast, unconstrained Bayesian approximation), and MEME (mixed-effects model evolution) to identify the statistically supported selection pressure sites. The analyses were conducted using the datamonkey adaptive evolution server (https://www.datamonkey.org/; accessed on 22 December 2022). Statistical support for positive and negative selection sites was defined as amino acid residues with a *p*-value of <0.1 for SLAC and MEME or a posterior probability of >0.9 for FUBAR. Notably, only the active coding region (of the same size, with the exception of the SEOV S gene) of each gene segment was included in the selective pressure analysis (Table 2).

### 2.5. Structural Analysis of the Gn Ectodomains of SEOV and HTNV

We compared the genetic homology of SEOV and HTNV by assessing the topology of the Gn glycoprotein including putative N-linked glycosylation patterns using online bioinformatic tools (https://services.healthtech.dtu.dk/; accessed on 3 March 2023). The Gn head (Gn^H^) ectodomains of the HTNV strain Aa19-233/2019 and SEOV strain GZRn134/2019 were reconstructed based on the prototypic Hantaan virus Gn^H^ (PDB: 7NRH [20]) using the comparative modeling of the protein three-dimensional structure prediction method (https://salilab.org/modeller/; accessed on 10 January 2023). The structural properties at the Gn^H^ ectodomain surface were assessed using UCSF Chimera X from the Resource for Biocomputing, Visualization, and Informatics (San Francisco, CA, USA), and the models were visualized using PyMOL Molecular Graphics System v2.1.0 (Schrödinger, Inc., New York, NY, USA).

## 3. Results

### 3.1. Phylogenetic Relationships of the Gene Segments of SEOV and HTNV

**Figure 1:**
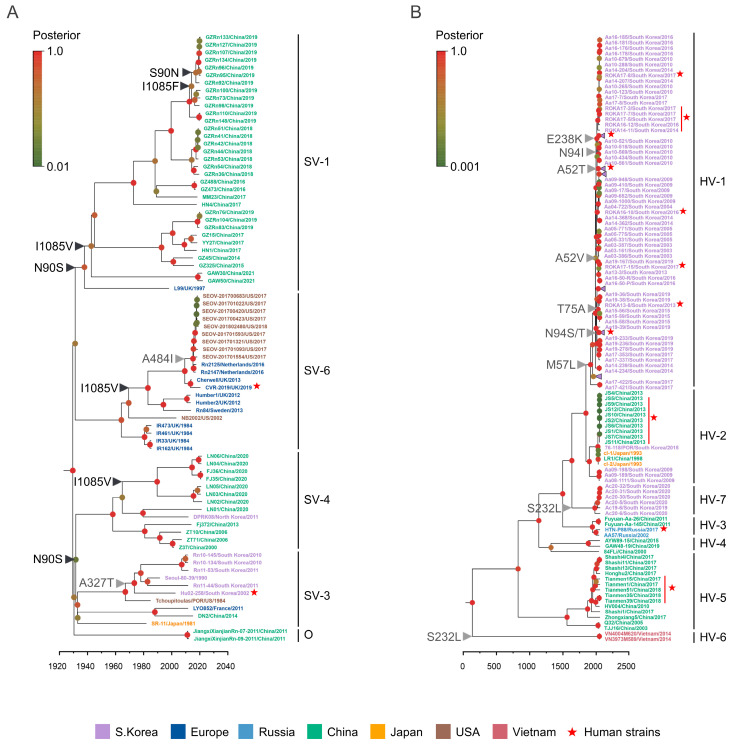
Phylogenetic analysis of SEOV and HTNV M gene segments. The maximum clade credibility trees of the full-length M genes of SEOV ((**A**); 3399 bps) and HTNV ((**B**); 3405 bps). The strains were color coded by their country of origin. Human strains are marked with red stars. Persistent sites under positive selective pressure are indicated with black arrowheads (FUBAR method), while gray arrowheads represent transient branch mutations (MEME method) (refer to Table 2).

In this study, we employed datasets comprising 80 complete genomes of SEOV and 146 complete genomes of HTNV, each covering the L, M, and S segments (Supplementary Table S1). As illustrated in Figure 1A, SEOV exhibited a wide distribution across Europe, North America, and Southeast Asia, with its most significant presence in China. In contrast, HTNV strains were predominantly collected in Eastern Asia, encompassing China, South Korea, and Japan including Russia and Vietnam (Figure 1B). Then, we reconstructed the phylogenetic relationships for the L, M, and S segments for each virus (Supplementary Figures S1 and S2). Genetic clades were defined based on the phylogenetic trees of the M segment obtained from previous investigations [8,10]. Among the 80 SEOV strains, there were two human strains, one from the United Kingdom (UK) and another from South Korea. For HTNV, 26 human strains out of 146 HTNV strains were mainly identified in China and South Korea.

Our analysis of the SEOV phylogenies revealed four major genetic clades (SV-1, SV-3, SV-4, and SV-6) of SEOV and one outlier (O) (Figure 1A). Clades SV-1 and SV-4 predominantly comprised strains from China including one from the U.K. in clade SV-1 and one from North Korea in clade SV-4. Clade SV-3 mainly included South Korean strains and strains from China, Japan, France, and the United States of America (USA), whereas clade SV-6 consisted of strains from the USA and European countries (the UK, the Netherlands, and Sweden). Notably, clade SV-1 shared a common ancestor with clade SV-6, while SV-3 shared its ancestry with SV-4.

For the M gene phylogeny of HTNV (Figure 1B), there were seven distinct genetic clades, denoted as HV-1 to HV-7. Clade HV-1 primarily contained strains from South Korea, spanning from 2003 to 2019. Clade HV-2 included strains from China, Japan, and South Korea. Clades HV-4 and HV-5 included viral strains isolated in China, while HV-3 contained strains from both China and Russia. Additionally, we identified two distinct genetic lineages, clades HV-6 and HV-7, comprising HTNVs isolated in Vietnam in 2014 and South Korea from 2019 to 2020, respectively.

Based on the phylogenetic relationships of the three gene segments of both viruses, we observed that some SEOVs retained the gene segments of heterogeneous clades, while almost all HTNVs possessed the same clade gene segments (Supplementary Figures S1 and S2). Notably, two SEOVs (JiangxiXianjianRn-07-2011/China/2011 and JiangxiXianjianRn-09-2011/China/2011) of clade O appeared to retain the L and S genes of clades SV-3 and SV-4, respectively. Also, LYO852/France/2011 and DN2/China/2014 of clade SV-3 retained the S gene of clade SV-1, and L99/UK/1997 and YY27/China/2017 of clade SV-1 were found to harbor the genes of clades SV-3 and SV-6 L, respectively. Especially, two 2021 SEOVs isolated in China (GAW30/China/2021 and GAW30/China/2021) of clade SV-1 harbored the S gene of clade SV-6, but their L genes appeared to be genetically distinct from other SV-1, -3, -4, and -6 L genes (Supplementary Figure S1). In the case of HTNVs, clades HV-1, -2, -3, -4, and -7 largely shared common ancestors in the M gene tree. However, in the L and S genes, clade HV-7 shared common ancestors with clade HV-6. These findings might suggest different evolutionary restrictions on genetic reassortment events between SEOV and HTNV.

### 3.2. Genetic Reassortment in SEOV and HTNV

**Figure 2:**
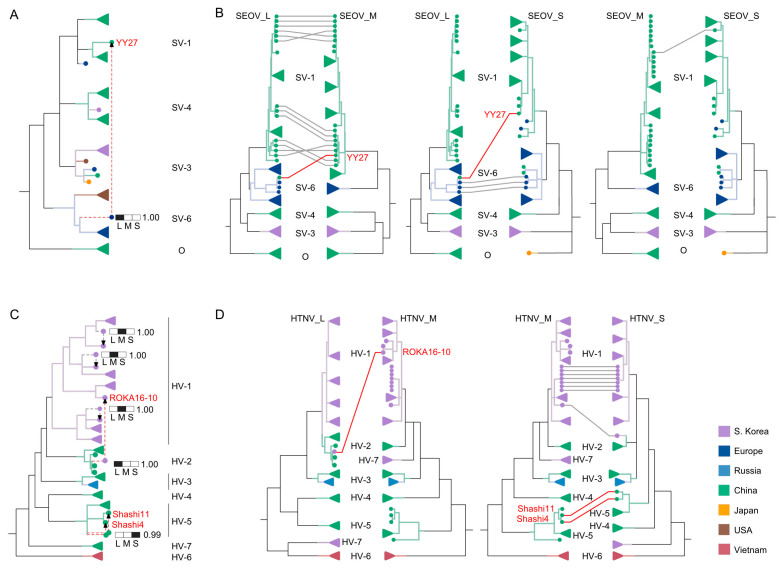
Detection of potential genetic reassortment in SEOV and HTNV. The genetic reassortment events within SEOV (**A**,**B**) and HTNV (**C**,**D**) were assessed using two distinct methods. The Bayesian analysis method, CoalRe, evaluates the probability distribution among all three segments in the reassortment network (**A**,**C**) (confidence level of greater than 0.9). The gene segment likely obtained from a different lineage is represented by a black square. The second method (GiRaF), based on Markov Chain Monte Carlo (MCMC) sampled trees, identifies incompatible splits in maximum likelihood (ML) trees for the L, M, and S segments (**B**,**D**). Events confirmed by both methods were highlighted in red. Dashed lines in (**A**,**C**) represent segments descending from different lineages. Colors for each clade branch represent the country of origin, as shown in Figure 1. For simplicity and enhanced visualization, phylogenetic branches without strains exhibiting reassortment were collapsed.

Our analysis unveiled distinct patterns of potential genetic reassortment within SEOV and HTNV, along with variations in the involved gene segments (Figure 2). Among the 80 SEOVs and 146 HTNVs, 18 (22.50%) and 13 (8.90%) were identified as putative reassortant viruses, respectively (listed in Supplementary Tables S2 and S3). Reassortment events were predominantly observed among SEOVs from clades SV-1 and SV-6 (Figure 2A,B). Conversely, within HTNV, reassortment events were mainly detected from clades HV-1 and HV-5 viruses (Figure 2C,D). Importantly, we confirmed that YY27/China/2017 of clade SV-1 SEOV resulted from the reassortment event(s) involving SV-1 L segment and SV-4 M and S segments (Figure 2A,B, and Supplementary Table S2). Similarly, ROKA16-10/South Korea/2016 of clade HV-1 HTNV underwent a similar reassortment process, involving the L gene segment from clade HV-2 HTNV (mainly isolated in China) and the M and S segments from clade HV-1 HTNV (isolated in South Korea), as corroborated by both CoelRe and GiRaF methods (Figure 2C,D, and Supplementary Table S3).

The M gene segment appeared to play a pivotal role in reassortment in both viruses, constituting 78.95% of SEOV and 76.92% of HTNV putative reassortments. However, SEOV demonstrated reassortment preference involving the L or M segment, while HTNV primarily showed exchange M or S segment (Figure 2A,C). These findings highlight the reassortment complexity and the different genetic compatibilities between SEOV and HTNV.

### 3.3. Genetic Diversity and Selective Pressure in SEOV and HTNV

**Figure 3:**
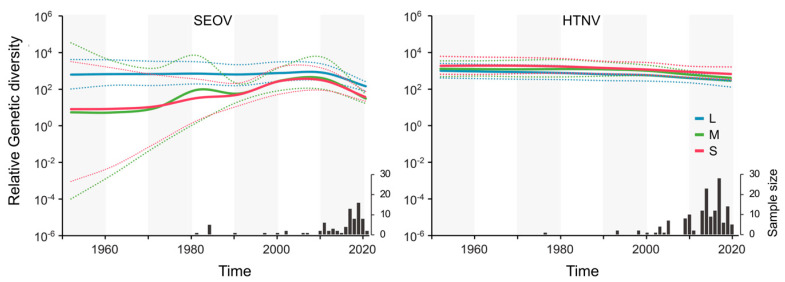
Genetic diversity comparison in L, M, and S gene segments of SEOV and HTNV. Bayesian Skygrid plots depict population size changes over time for SEOV and HTNV across L (light blue line), M (green line), and S (red line) gene segments. The areas within dotted lines represent 95% highest and lowest posterior density intervals, indicating data uncertainty levels.

As illustrated with the Skygrid plots in Figure 3, our analysis revealed the dynamic patterns of genetic diversity and the selective pressure within SEOV and HTNV. Specifically, a continuous and gradual decrease in genetic diversity across all HTNV gene segments from 1980 to 2022 was observed. In contrast, SEOV displayed a distinct pattern, with the L segment resembling HTNV, while the M and S segments consistently exhibited an increasing genetic diversity between 1980 and 2010, contrasting the corresponding HTNV segments. Notably, SEOV M and S gene mutation rates (11.7 × 10^−4^ and 11.2 × 10^−4^ sub./site/year, respectively) surpassed those of HTNV (Table 1). The most recent common ancestor for SEOV M and S gene segments was estimated in 1959 and 1961, respectively, in contrast to over a millennium ago for HTNV.

The analysis of natural selection unveiled a prevalent purifying selection acting on both viruses throughout their evolutionary histories, as detailed in Table 2. Notably, HTNV Gn exhibited a higher dN/dS (ω) ratio, compared to Gc, while in SEOV, the Gc showed a higher ω value than the Gn ectodomain. Employing the MEME method, we identified eleven positively selected sites in HTNV Gn domain and four in SEOV. Interestingly, the FUBAR method indicated only one positively selected site in SEOV Gn (residue 90) and Gc (residue 1085) domains, with none observed in the corresponding HTNV gene segments. Remarkably, we consistently observed mutations like N90S and I1085V along the entire phylogeny of the SEOV M segment (Figure 1A), underscoring the significance of these sites in the evolutionary dynamics of SEOV.

Notably, mutations in SEOV, such as A327T in SV-3 (associated with South Korea) and A484I in SV-6 (predominantly observed in the USA), may also be under positive selection within specific geographic regions (Figure 1A, marked in gray and Table 2). Furthermore, amino acid mutations at residues 52, 57, 94, and 232, with a transient effect on selective pressure, displayed recurrent appearances and may have played a pivotal role in the host–virus co-evolution, based on the geographic origin along the HTNV phylogeny (Figure 1B).

### 3.4. Structural Landscape of SEOV and HTNV Gn Ectodomains

**Figure 4:**
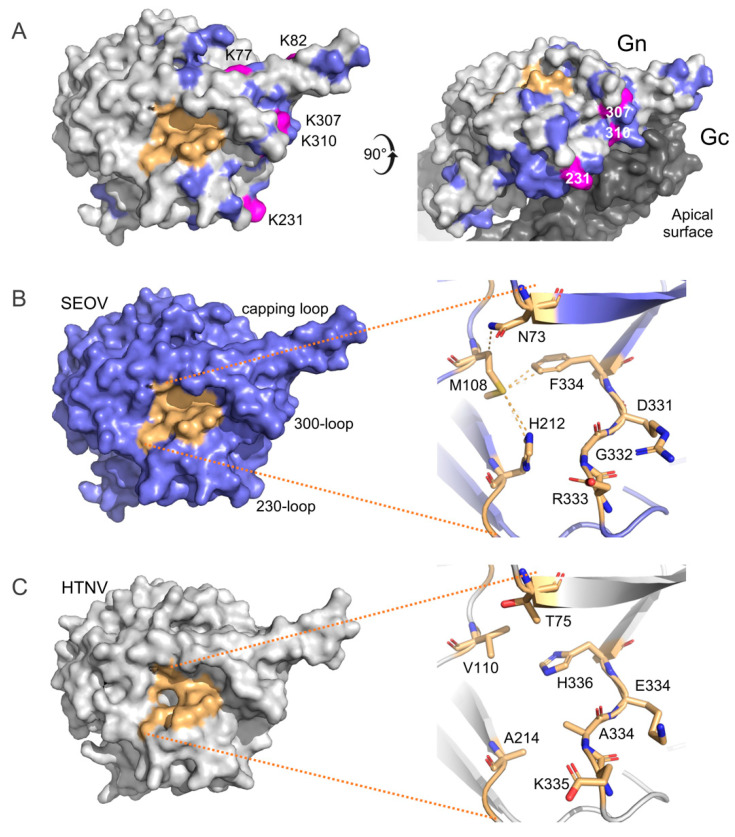
Comparative molecular analysis of HTNV and SEOV Gn ectodomains. (**A**) Distinct surface amino acid residues specific to the SEOV Gn ectodomain (highlighted in blue), differing from HTNV Gn (in gray), are depicted. Specific lysine (K) residues potentially forming a positively charged ‘lysine fence’ within crucial surface motifs are indicated in magenta. (**B**) Molecular structure of SEOV Gn and (**C**) HTNV Gn, with an emphasis on the pocket-like configuration (highlighted in orange), thereby exposing the amino acid differences and similarities between the two viruses (shown on the right). Expected physical interactions within the SEOV Gn pocket are visualized at a resolution of 3.5–4.0 Å.

Considering the observed differences in genetic diversity and selective pressures within SEOV and HTNV M genes, we conducted a comparative analysis of their Gn ectodomains. Linear M gene sequences from SEOV and HTNV exhibited similar glycoprotein precursor topologies and putative N-linked glycosylation patterns (Supplementary Table S4). Our comparison of the linear and simulated three-dimensional (3D) structures of SEOV and HTNV Gn revealed 73 differing amino acids (Supplementary Table S5). Among these, 40 residues were located at the apical surface of Gn (Figure 4A), and 9 were within the pocket-like motif (Figure 4B,C). Intriguingly, our analysis indicated potential flexibility in the 190-helix and potentially a greater positively charged apical surface in SEOV Gn (Supplementary Figure S3). Upon further examination, we identified five lysine (K) residues, with three located within the 230- and 300-loops (K231, K307, and K310) on the apical surface of SEOV Gn and two in the capping loop (K77 and K82), distinguishing them from those of HTNV (Figure 4A and Supplementary Table S5). Notably, these amino acid positions were under a purifying selective pressure and showed conservation in SEOV (Table 2 and Table S2), potentially contributing to the increased positive charge on the Gn surface.

Moreover, we found structural differences in the pocket-like motif between SEOV and HTNV Gn. In SEOV Gn, the side chain of residue M108 exhibited a closer proximity to residues N73, H212, and particularly the aromatic ring of residue F334 (3.5–4.0 Å) (Figure 4B), while HTNV Gn featured shorter side chains in the pocket (Figure 4C). Additionally, SEOV Gn harbored amino acid residues D331, G332, and R333 (Supplementary Figure S3) adjacent to F334, forming a tripeptide motif ‘RGD’ known to be recognized by many integrins [7,21]. Intriguingly, HTNV Gn contained residues E334, A335, and K336, forming a functionally similar cluster at the same position when compared to SEOV. These findings highlight both structural differences and similarities within the Gn of SEOV and HTNV, offering insights into viral antigenic structures and potential viral adaptations.

## 4. Discussion

In this study, we investigated the complete genome sequences to enhance our understanding of the evolutionary dynamics of the closely related SEOV and HTNV. By conducting a comparative analysis of their complete genome sequences, we identified distinct phylogenetic clades within both viruses. The substantial increase in genetic diversity observed in SEOV is particularly noteworthy, especially in the M and S gene segments, accompanied by higher mutation rates (11.7 × 10^−4^ and 11.2 × 10^−4^ substitutions per site per year, respectively), suggesting rapid evolution, compared to HTNV. In contrast, the L segment in both viruses maintained higher and more consistent population diversity and evolutionary rates. Additionally, SEOV L, M, and S segments exhibited a stronger phylogenetic support and a more recent origin compared to their HTNV counterparts. These differences in genetic diversity and evolutionary patterns could be attributed to SEOV’s global distribution facilitated by its carrier hosts, i.e., rats, through global trade, as well as the intricate co-evolution of orthohantaviruses with specific rodent species [22]. Furthermore, recent molecular confirmations of SEOV in rats across Africa [4], the Americas [23], and Europe [12,24] emphasize its substantial importance as a public health concern.

The disparities in phylogenetic positions within major genetic clades that we identified (Supplementary Figures S1 and S2) likely result from distinct evolutionary pathways, including previous genetic reassortment events [25]. Despite the relatively infrequent occurrence of reassortment in both viruses, the variations in reassortment dynamics, such as SEOV preference for L or M segment exchange and HTNV inclination toward M or S segment reassortment, may also be associated with HTNV’s regional distribution and SEOV’s global spread. Notably, two particular viral strains, YY27/China/2017 of SEOV and ROKA 16–10/South Korea/2016 of HTNV, displayed clear compatibility between the M and S segments of one genetic clade with the L segment from another (Figure 2). Our findings align with prior research indicating the potential for genetic reassortment involving the L segment in HTNV [9] as well as the hypothesis that the M segment exchange may be potentially more tolerable or even beneficial in orthohantaviruses [26]. Furthermore, the instances of hybrid viruses containing the L and M segments from SEOV and the S segment from HTNV have been documented [27]. This again emphasizes the intricate nature of genetic reassortment in HFRS viruses, its potential complexity and versatility, and the more recent origin of SEOV and highlights the need for further investigation, particularly on a broader scale.

In our analysis of natural selection, both SEOV and HTNV predominantly exhibited purifying selection, although distinctive dN/dS ratios and variations in positively selected residues were observed, as summarized in Table 2. Interestingly, HTNV Gn ectodomain showed a higher dN/dS ratio of 0.042, while SEOV dN/dS ratio displayed an increase from Gn to Gc, reaching 0.041. These differences were reflected in the number of positively selected amino acid positions in the Gn and Gc regions of SEOV and HTNV. These distinctive patterns of selective pressures acting on viral genomes provide valuable insights into the molecular determinants that influence the divergent virus–host co-evolutionary trajectories of SEOV and HTNV.

The Gn ectodomain has emerged as a pivotal target for the host’s immune response, potentially establishing it as the dominant immunogenic region, compared to Gc [20,28]. Our investigation highlighted three surface-exposed regions situated at the apex of the Gn protein (around amino acid positions 230 and 300 and within the capping loop). These regions could contribute to structural differences, the emergence of escape mutations, and antigenic distinctions among hantaviruses [29]. Among the amino acid residues distinguishing the Gn ectodomains of the two viruses, 40 residues were located on the surface, projecting outward and likely influencing their antigenic characteristics (Figure 4A). Importantly, these residues might be specific for each virus [29] and, as our results indicated, likely maintained under purifying selection (Table 2). Additionally, the five specific lysine residues (K77, K82, K231, K307, and K310 in SEOV Gn) on the surface may collectively influence the antigenic properties, potentially forming a positively charged ‘lysine fence’ [30,31]. This structural difference may contribute to distinct immunological responses and explain instances of misdiagnosis, compared to HTNV [32,33].

While the relationship between the recognition of α_V_β3 integrin and human HFRS by viruses remains enigmatic [7], our study elucidated specific disparities and similarities in SEOV and HTNV Gn ectodomain structures (Figure 4). Within SEOV Gn, the residue M106, situated in the pocket, possesses a long and flexible side chain that may interact with various amino acids, including F334, forming a Met-Aro bridge that may potentially stabilize receptor-binding motifs [34]. Notably, HTNV Gn putative pocket exhibits shorter hydrophobic side-chain residues, suggesting a more flexible structure that could be potentially targeted by hydrophobic peptides [35]. Adjacent to F334 in SEOV Gn, we identified residues R333, G332, and D331, mirroring HTNV Gn K336, A335, and E334, which indicates functional similarity. The presence of an RGD-like motif, recognized by multiple integrins [7], prompts the exploration of its functional significance in HFRS viruses. Its proximity to the stable Met-Aro bridge in SEOV Gn spikes motivates comparative integrin receptor binding research, which is vital for understanding infection mechanisms and the design of potential therapeutics.

SEOV-associated human infections are characterized by nonspecific clinical features [36], often leading to misdiagnosis or delayed identification, compared to HTNV [32,33], suggesting transient and often chameleon-like zoonotic symptoms. Nevertheless, knowledge gaps persist regarding SEOV and HTNV interactions with endothelial and immune cells, as well as their interplay with platelets [37]. Thus, further investigations are warranted to discern the precise physical interactions. Although not directly tied to public health outcomes, the insights into the evolutionary dynamics of SEOV and HTNV can inform surveillance and monitoring efforts. Collaborative efforts to collect and share high-quality genomic sequences will be crucial in staying ahead of these medically significant viruses.

Here, we investigated the intricate evolutionary dynamics of SEOV and HTNV. The distinctive patterns of genetic diversity, reassortment, and selective pressures observed between these two viruses underscore the need for continued research into the molecular determinants that drive their distinct virus–host co-evolutionary trajectories. Additionally, our findings highlight the potential significance of specific viral protein residues in influencing antigenicity and integrin receptor binding, offering promising avenues for further investigation and the development of targeted medical interventions. Ultimately, a deeper understanding of these viruses’ biology and evolution is essential for effective surveillance and control measures to mitigate their impact on public health.

## Figures and Tables

**Table 1:** Bayesian estimates of evolutionary rates and times to the most recent common ancestor (tMRCA) for SEOV and HTNV.

Virus (Strains)	Gene Segment	Size (nt)	Substitution/Site/Year, 10^−4^ (95% HPD)	tMRCA (Year, 95% HPD)
SEOV (80)	L	6288	2.07 (0.837–3.67)	1094 (57–1756)
	M	3399	11.7 (5.68–21.9)	1959 (1933–1977)
	S	939 *	11.2 (5.32–17.8)	1961 (1937–1977)
HTNV (146)	L	6453	3.65 (1.69–5.58)	>1000
	M	3405	1.96 (1.14–2.90)	>1000
	S	1287	1.18 (0.37–2.0)	>1000

* The S segment sequences were trimmed because of truncated N-terminus in 22 SEOV strains. tMRCA, time of the most recent common ancestor.

**Table 2:** Comparative analysis of amino acid positions under selective pressure in SEOV and HTNV.

Virus	Gene (Segment)	Start–Stop Position	dN/dS (ω)	SLAC			FUBAR			MEME	
				N	P	Sites	N	P	Sites	P	Sites
**SEOV**	RdRp (L)	562–1285	0.021	237	0	-	542	0	-	9	735, 753, 773, 810, 896, 1044, 1045, 1141, 1183
	Gn (M)	19–542	0.031	171	0	-	367	1	90	4	96, 327, 409, 484
	Gc (M)	627–1105	0.041	156	0	-	306	1	1085	8	674, 711, 809, 968, 1022, 1051, 1068, 1093
	NP (S)	117–429	0.030	82	0	-	183	0	-	3	334, 336, 369
**HTNV**	RdRp (L)	562–1285	0.020	566	0	-	687	0	-	14	582, 657, 699, 724, 739, 741, 743, 761, 787, 918, 1090, 1111, 1238, 1288
	Gn (M)	21–544	0.042	341	0	-	461	0	-	12	52, 57, 75, 94, 103, 173, 208, 232, 238, 282, 407
	Gc (M)	629–1108	0.028	331	0	-	419	0	-	7	635, 812, 818, 837, 991, 1021, 1039
	NP (S)	1–429	0.028	266	0	-	378	0	-	11	2, 3, 23, 32, 33, 50, 58, 63, 183, 250, 387

N, number of negative selected sites; P, number of positive selected sites; NP, nucleocapsid protein; RdRp, RNA-dependent RNA polymerase. Gn and Gc, the two ectodomains of the surface precursor glycoprotein encoded by M gene segment.

## Data Availability

All pertinent data are included in the manuscript or available upon request.

## Supplementary Materials

The following supporting information can be downloaded at: https://www.mdpi.com/article/10.3390/v16010105/s1, Table S1: Isolation region, collection year and host information for SEOV and HTNV; Table S2: List of reassortment events in SEOV; Table S3: List of reassortment events in HTNV; Table S4: Comparative features of linear consensus sequences for the M glycoprotein precursors of SEOV and HTNV; Table S5: Distinct amino acid residues in SEOV’s Gn compared to HTNV; Figure S1: Phylogenetic relationships of SEOV genome segments; Figure S2: Phylogenetic relationships of HTNV genome segments; Figure S3: Inferred reassortment events using GiRaF analysis for SEOV and HTNV; Figure S4: Structural properties of the SEOV & HTNV Gn ectodomains.
