# Supplementary material for: Exploring the Genetic Diversity and Molecular Evolution of Seoul and Hantaan Orthohantaviruses
Source: Viruses. 2024 Jan 11;16(1):105. doi: 10.3390/v16010105 (PMC10818986; doi:10.3390/v16010105)
Supplement: Supplementary file 1 [file viruses-16-00105-s001.zip › viruses-2789302-supplementary.pdf]

Supplementary material

Exploring the Genetic Diversity and Molecular Evolution of Seoul and Hantaan Orthohantaviruses

Atanas V. Demirev, Sangyi Lee, Sejik Park , Hyunbeen Kim, Seunghye Cho, Kyuyoung Lee, Kisoon Kim, Jin-Won Song, Man-Seong Park, and Jin Il Kim

Supplementary material includes:

Figures S1-S4

Tables S1-S5

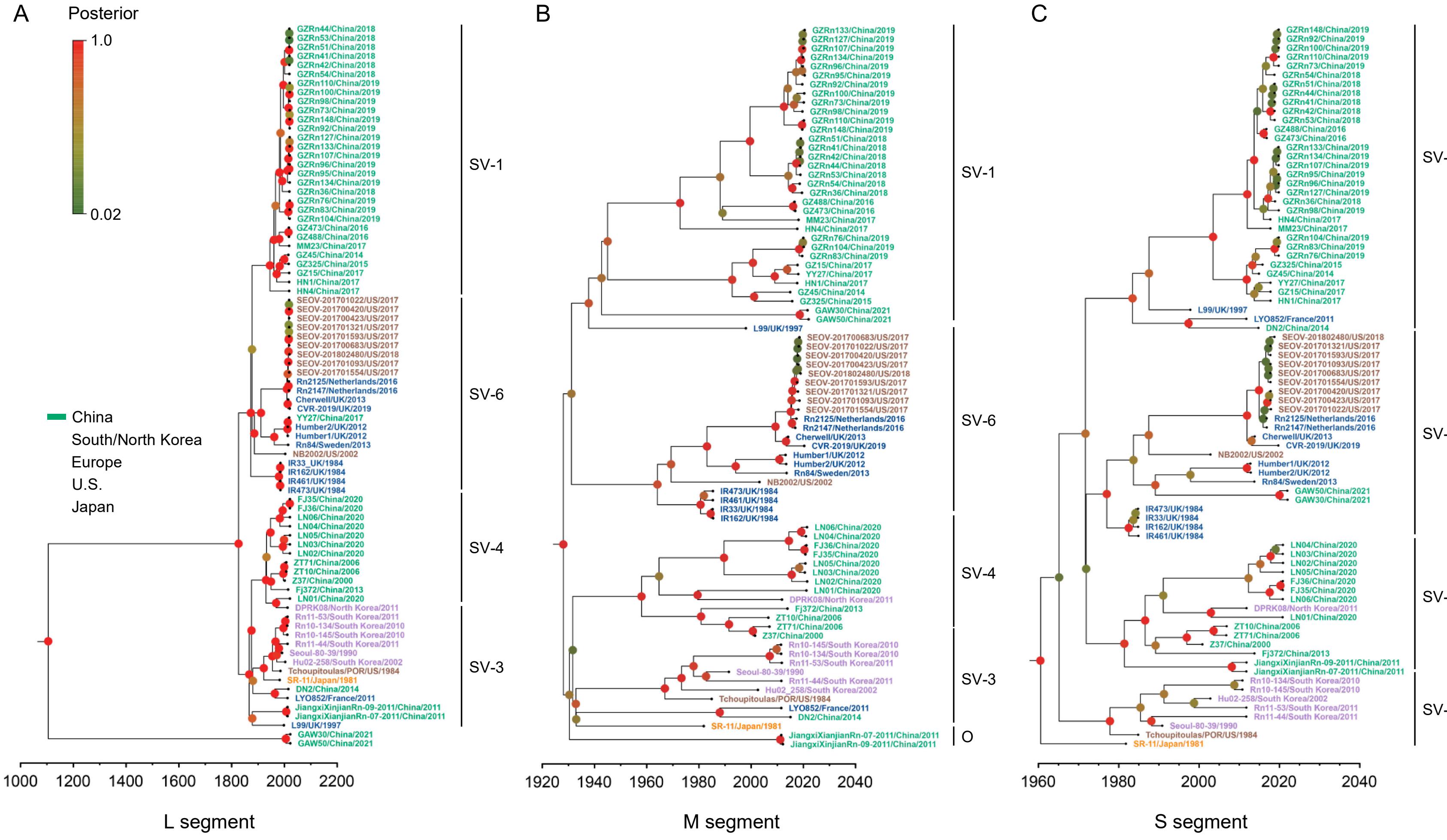

Figure S1. Phylogenetic relationships of SEOV genome segments.

Maximum Clade Credibility (MCC) trees for L (A), M (B), and S (C) segments of SEOV were reconstructed using Bayesian evolutionary inference. Strains were color-labeled by origin countries. The segment branches were categorized into clades SV-1, SV3, SV-4, SV-6 and outlier clade O based on previous lineage defining strains [7].

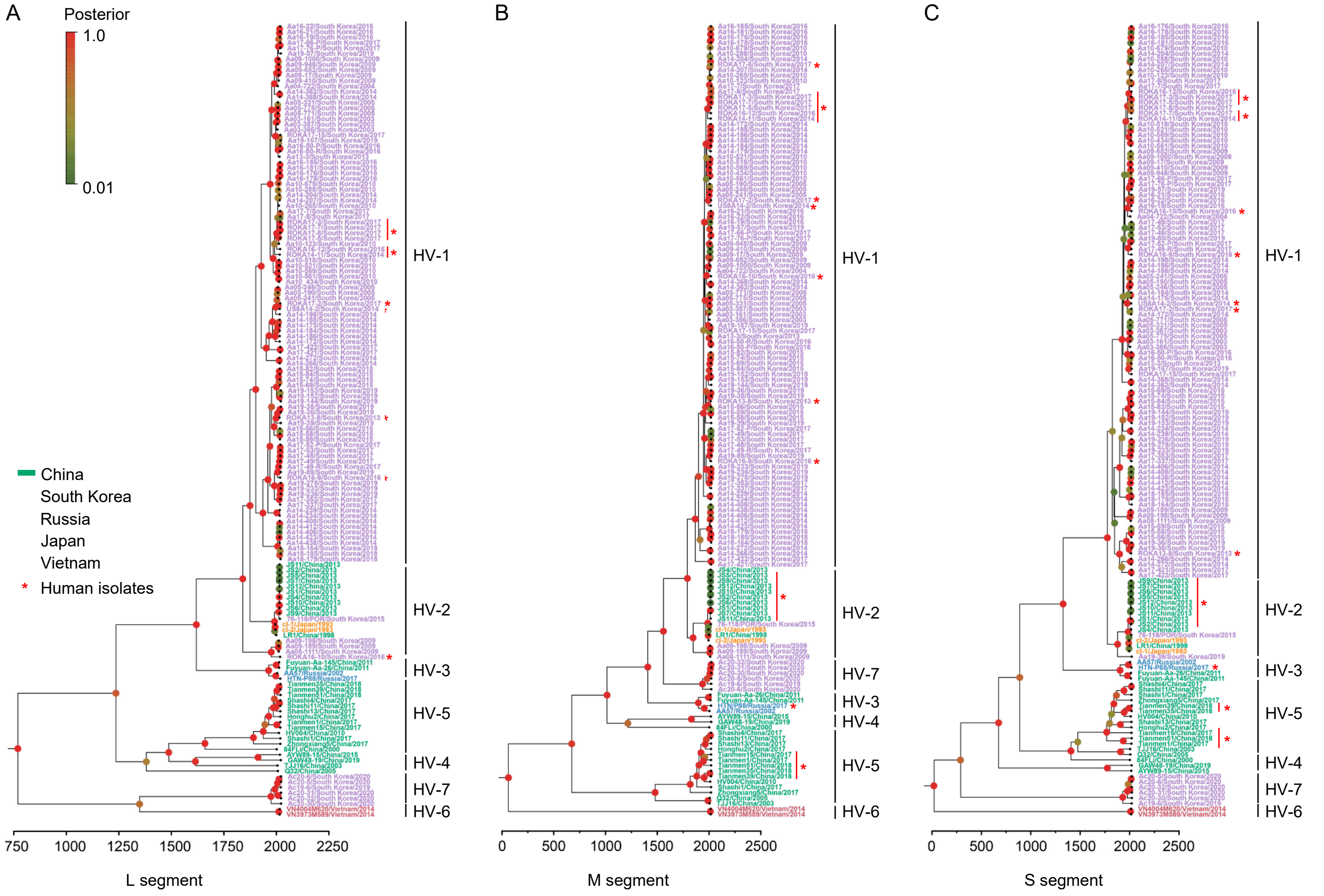

Figure S2. Phylogenetic relationships of HTNV genome segments.

Maximum Clade Credibility (MCC) trees for L (A), M (B), and S (C) segments of HTNV were reconstructed using Bayesian evolutionary inference. Strains were color-labeled by origin countries. The segment branches were categorized into clades HV-1 to HV-7 based on previously defined clades in M phylogenetic trees [9].

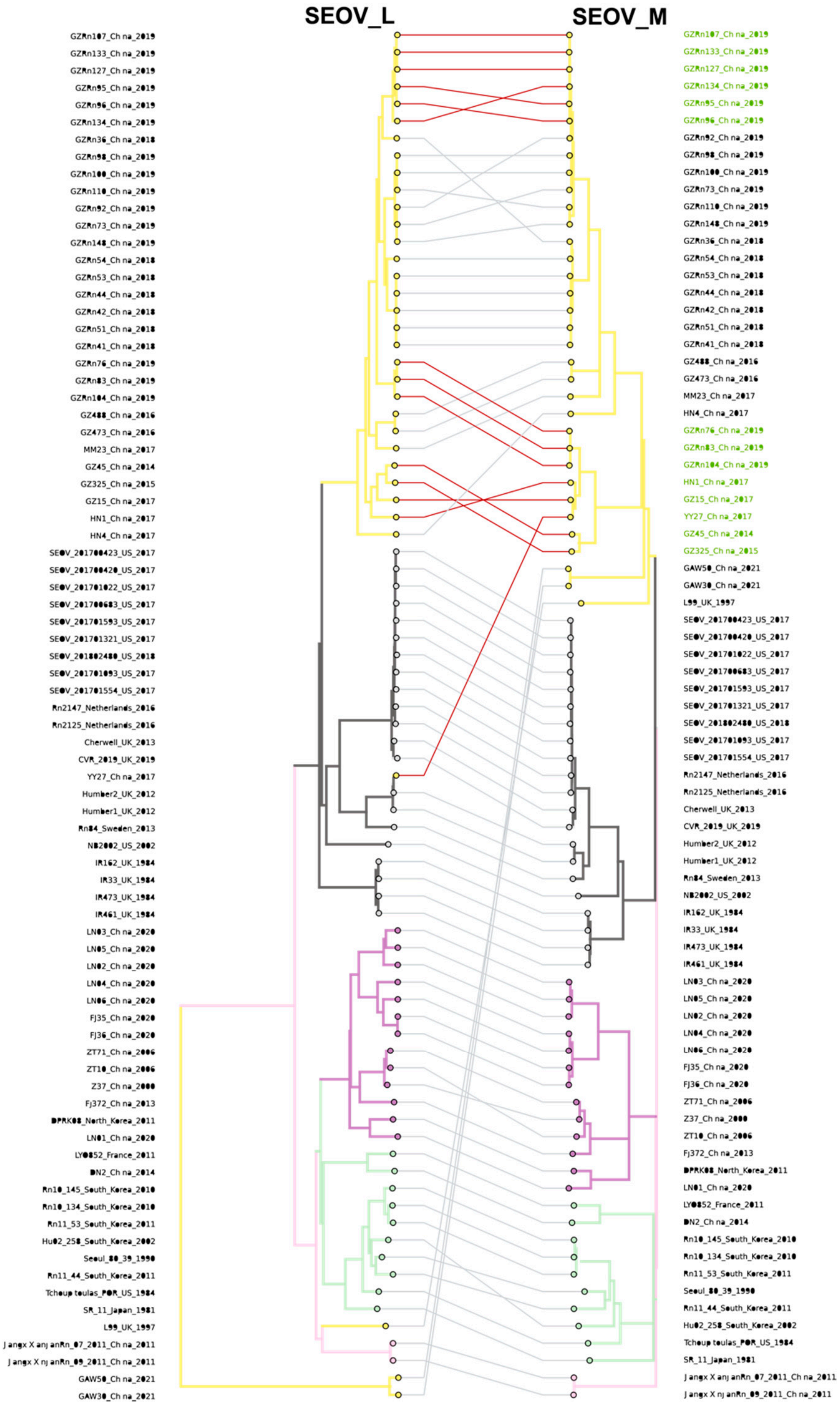

SEOV_L
SEOV_M
GZRn107_Ch na_2019
GZRn133_Ch na_2019
GZRn127_Ch na_2019
GZRn95_Ch na_2019
GZRn96_Ch na_2019
GZRn134_Ch na_2019
GZRn36_Ch na_2018
GZRn98_Ch na_2019
GZRn100_Ch na_2019
GZRn110_Ch na_2019
GZRn92_Ch na_2019
GZRn73_Ch na_2019
GZRn148_Ch na_2019
GZRn54_Ch na_2018
GZRn53_Ch na_2018
GZRn44_Ch na_2018
GZRn42_Ch na_2018
GZRn51_Ch na_2018
GZRn41_Ch na_2018
GZRn76_Ch na_2019
GZRn83_Ch na_2019
GZRn104_Ch na_2019
GZ488_Ch na_2016
GZ473_Ch na_2016
MM23_Ch na_2017
GZ45_Ch na_2014
GZ325_Ch na_2015
GZ15_Ch na_2017
HN1_Ch na_2017
HN4_Ch na_2017
SEOV_201700423_US_2017
SEOV_201700420_US_2017
SEOV_201701022_US_2017
SEOV_201700683_US_2017
SEOV_201701593_US_2017
SEOV_201701321_US_2017
SEOV_201802480_US_2018
SEOV_201701093_US_2017
SEOV_201701554_US_2017
Rn2147_Netherlands_2016
Rn2125_Netherlands_2016
Cherwell_UK_2013
CVR_2019_UK_2019
YY27_Ch na_2017
Humber2_UK_2012
Humber1_UK_2012
Rn84_Sweden_2013
NB2002_US_2002
IR162_UK_1984
IR33_UK_1984
IR473_UK_1984
IR461_UK_1984
LN03_Ch na_2020
LN05_Ch na_2020
LN02_Ch na_2020
LN04_Ch na_2020
LN06_Ch na_2020
FJ35_Ch na_2020
FJ36_Ch na_2020
ZT71_Ch na_2006
ZT10_Ch na_2006
Z37_Ch na_2000
Fj372_Ch na_2013
DPRK08_North_Korea_2011
LN01_Ch na_2020
LY0852_France_2011
DN2_Ch na_2014
Rn10_145_South_Korea_2010
Rn10_134_South_Korea_2010
Rn11_53_South_Korea_2011
Hu02_258_South_Korea_2002
Seoul_80_39_1990
Rn11_44_South_Korea_2011
Tchoup toulas_POR_US_1984
SR_11_Japan_1981
L99_UK_1997
J angx X anj anRn_07_2011_Ch na_2011
J angx X nj anRn_09_2011_Ch na_2011
GAW50_Ch na_2021
GAW30_Ch na_2021
GZRn107_Ch na_2019
GZRn133_Ch na_2019
GZRn127_Ch na_2019
GZRn134_Ch na_2019
GZRn95_Ch na_2019
GZRn96_Ch na_2019
GZRn92_Ch na_2019
GZRn98_Ch na_2019
GZRn100_Ch na_2019
GZRn73_Ch na_2019
GZRn110_Ch na_2019
GZRn148_Ch na_2019
GZRn36_Ch na_2018
GZRn54_Ch na_2018
GZRn53_Ch na_2018
GZRn44_Ch na_2018
GZRn42_Ch na_2018
GZRn51_Ch na_2018
GZRn41_Ch na_2018
GZ488_Ch na_2016
GZ473_Ch na_2016
MM23_Ch na_2017
HN4_Ch na_2017
GZRn76_Ch na_2019
GZRn83_Ch na_2019
GZRn104_Ch na_2019
HN1_Ch na_2017
GZ15_Ch na_2017
YY27_Ch na_2017
GZ45_Ch na_2014
GZ325_Ch na_2015
GAW50_Ch na_2021
GAW30_Ch na_2021
L99_UK_1997
SEOV_201700423_US_2017
SEOV_201700420_US_2017
SEOV_201701022_US_2017
SEOV_201700683_US_2017
SEOV_201701593_US_2017
SEOV_201701321_US_2017
SEOV_201802480_US_2018
SEOV_201701093_US_2017
SEOV_201701554_US_2017
Rn2147_Netherlands_2016
Rn2125_Netherlands_2016
Cherwell_UK_2013
CVR_2019_UK_2019
Humber2_UK_2012
Humber1_UK_2012
Rn84_Sweden_2013
NB2002_US_2002
IR162_UK_1984
IR33_UK_1984
IR473_UK_1984
IR461_UK_1984
LN03_Ch na_2020
LN05_Ch na_2020
LN02_Ch na_2020
LN04_Ch na_2020
LN06_Ch na_2020
FJ35_Ch na_2020
FJ36_Ch na_2020
ZT71_Ch na_2006
Z37_Ch na_2000
ZT10_Ch na_2006
Fj372_Ch na_2013
DPRK08_North_Korea_2011
LN01_Ch na_2020
LY0852_France_2011
DN2_Ch na_2014
Rn10_145_South_Korea_2010
Rn10_134_South_Korea_2010
Rn11_53_South_Korea_2011
Seoul_80_39_1990
Rn11_44_South_Korea_2011
Hu02_258_South_Korea_2002
Tchoup toulas_POR_US_1984
SR_11_Japan_1981
J angx X anj anRn_07_2011_Ch na_2011
J angx X nj anRn_09_2011_Ch na_2011

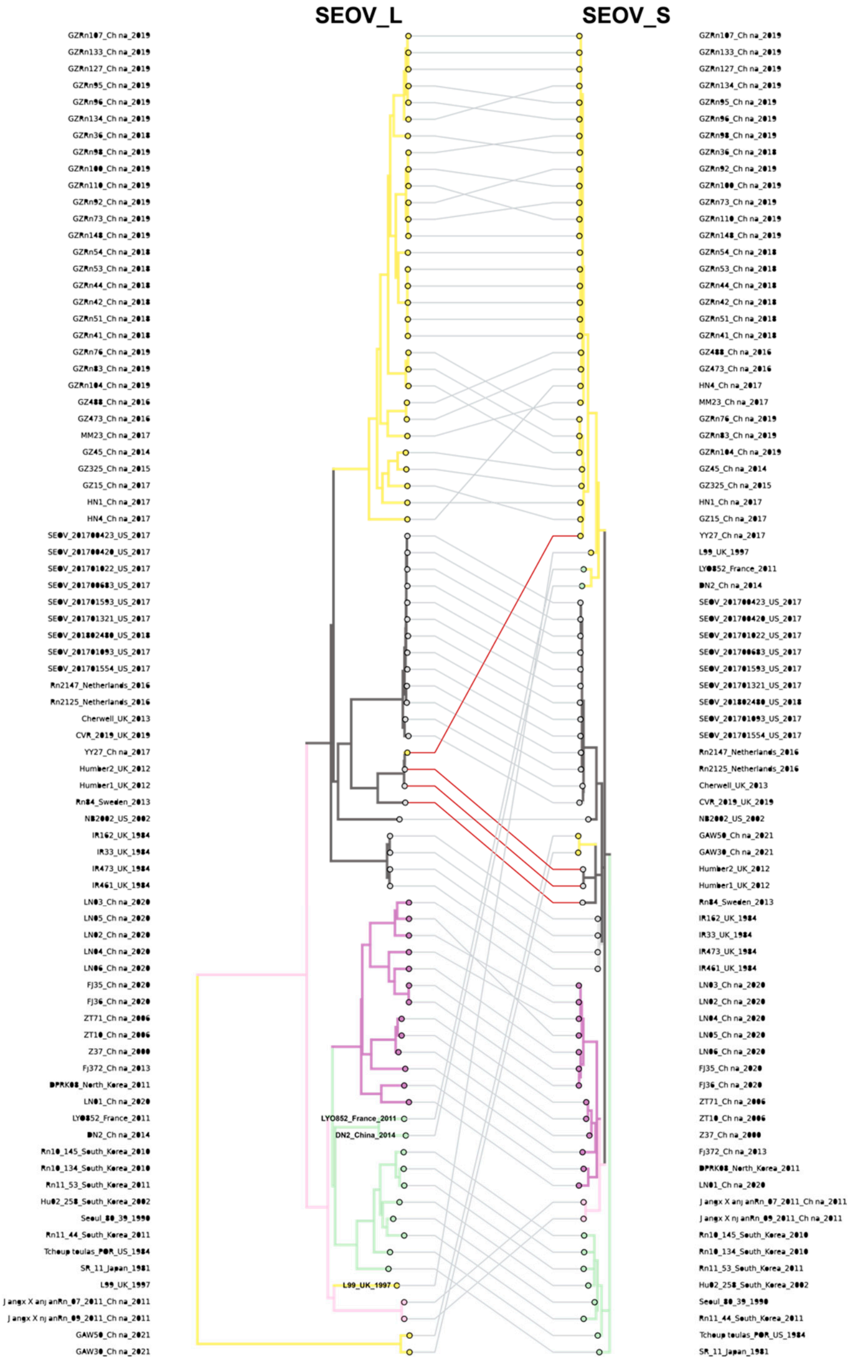

SEOV_L
SEOV_S
GZRn107_Ch na_2019
GZRn133_Ch na_2019
GZRn127_Ch na_2019
GZRn95_Ch na_2019
GZRn96_Ch na_2019
GZRn134_Ch na_2019
GZRn36_Ch na_2018
GZRn98_Ch na_2019
GZRn100_Ch na_2019
GZRn110_Ch na_2019
GZRn92_Ch na_2019
GZRn73_Ch na_2019
GZRn148_Ch na_2019
GZRn54_Ch na_2018
GZRn53_Ch na_2018
GZRn44_Ch na_2018
GZRn42_Ch na_2018
GZRn51_Ch na_2018
GZRn41_Ch na_2018
GZRn76_Ch na_2019
GZRn83_Ch na_2019
GZRn104_Ch na_2019
GZ488_Ch na_2016
GZ473_Ch na_2016
MM23_Ch na_2017
GZ45_Ch na_2014
GZ325_Ch na_2015
GZ15_Ch na_2017
HN1_Ch na_2017
HN4_Ch na_2017
SEOV_201700423_US_2017
SEOV_201700420_US_2017
SEOV_201701022_US_2017
SEOV_201700683_US_2017
SEOV_201701593_US_2017
SEOV_201701321_US_2017
SEOV_201802480_US_2018
SEOV_201701093_US_2017
SEOV_201701554_US_2017
Rn2147_Netherlands_2016
Rn2125_Netherlands_2016
Cherwell_UK_2013
CVR_2019_UK_2019
YY27_Ch na_2017
Humber2_UK_2012
Humber1_UK_2012
Rn84_Sweden_2013
NB2002_US_2002
IR162_UK_1984
IR33_UK_1984
IR473_UK_1984
IR461_UK_1984
LN03_Ch na_2020
LN05_Ch na_2020
LN02_Ch na_2020
LN04_Ch na_2020
LN06_Ch na_2020
FJ35_Ch na_2020
FJ36_Ch na_2020
ZT71_Ch na_2006
ZT10_Ch na_2006
Z37_Ch na_2000
Fj372_Ch na_2013
DPRK08_North_Korea_2011
LN01_Ch na_2020
LY0852_France_2011
DN2_Ch na_2014
Rn10_145_South_Korea_2010
Rn10_134_South_Korea_2010
Rn11_53_South_Korea_2011
Hu02_258_South_Korea_2002
Seoul_80_39_1990
Rn11_44_South_Korea_2011
Tchoup toulas_POR_US_1984
SR_11_Japan_1981
L99_UK_1997
J angx X anj anRn_07_2011_Ch na_2011
J angx X nj anRn_09_2011_Ch na_2011
GAW50_Ch na_2021
GAW30_Ch na_2021
LY0852_France_2011
DN2_China_2014
L99_UK_1997
GZRn107_Ch na_2019
GZRn133_Ch na_2019
GZRn127_Ch na_2019
GZRn134_Ch na_2019
GZRn95_Ch na_2019
GZRn96_Ch na_2019
GZRn98_Ch na_2019
GZRn36_Ch na_2018
GZRn92_Ch na_2019
GZRn100_Ch na_2019
GZRn73_Ch na_2019
GZRn110_Ch na_2019
GZRn148_Ch na_2019
GZRn54_Ch na_2018
GZRn53_Ch na_2018
GZRn44_Ch na_2018
GZRn42_Ch na_2018
GZRn51_Ch na_2018
GZRn41_Ch na_2018
GZ488_Ch na_2016
GZ473_Ch na_2016
HN4_Ch na_2017
MM23_Ch na_2017
GZRn76_Ch na_2019
GZRn83_Ch na_2019
GZRn104_Ch na_2019
GZ45_Ch na_2014
GZ325_Ch na_2015
HN1_Ch na_2017
GZ15_Ch na_2017
YY27_Ch na_2017
L99_UK_1997
LY0852_France_2011
DN2_Ch na_2014
SEOV_201700423_US_2017
SEOV_201700420_US_2017
SEOV_201701022_US_2017
SEOV_201700683_US_2017
SEOV_201701593_US_2017
SEOV_201701321_US_2017
SEOV_201802480_US_2018
SEOV_201701093_US_2017
SEOV_201701554_US_2017
Rn2147_Netherlands_2016
Rn2125_Netherlands_2016
Cherwell_UK_2013
CVR_2019_UK_2019
NB2002_US_2002
GAW50_Ch na_2021
GAW30_Ch na_2021
Humber2_UK_2012
Humber1_UK_2012
Rn84_Sweden_2013
IR162_UK_1984
IR33_UK_1984
IR473_UK_1984
IR461_UK_1984
LN03_Ch na_2020
LN02_Ch na_2020
LN04_Ch na_2020
LN05_Ch na_2020
LN06_Ch na_2020
FJ35_Ch na_2020
FJ36_Ch na_2020
ZT71_Ch na_2006
ZT10_Ch na_2006
Z37_Ch na_2000
Fj372_Ch na_2013
DPRK08_North_Korea_2011
LN01_Ch na_2020
J angx X anj anRn_07_2011_Ch na_2011
J angx X nj anRn_09_2011_Ch na_2011
Rn10_145_South_Korea_2010
Rn10_134_South_Korea_2010
Rn11_53_South_Korea_2011
Hu02_258_South_Korea_2002
Seoul_80_39_1990
Rn11_44_South_Korea_2011
Tchoup toulas_POR_US_1984
SR_11_Japan_1981

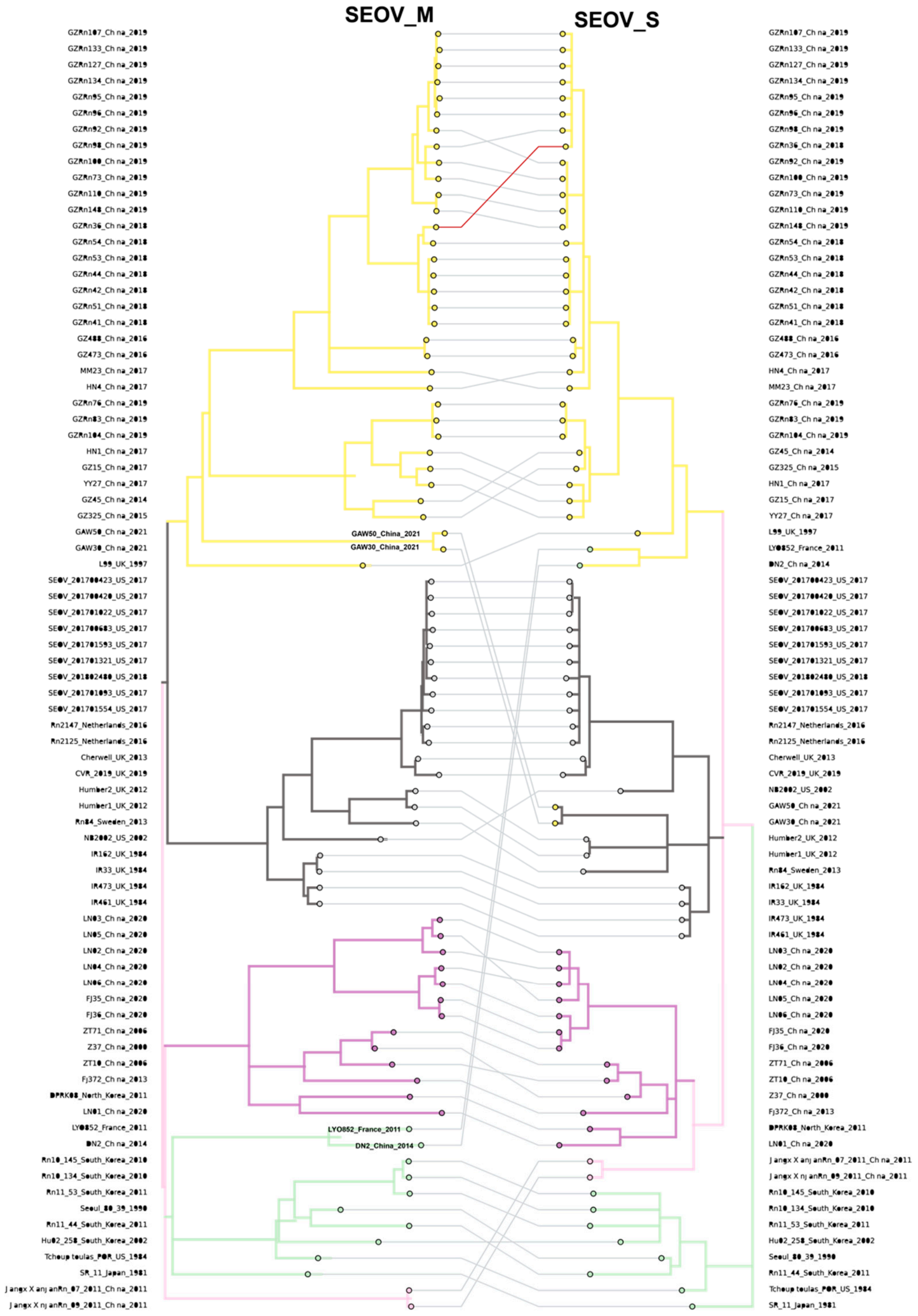
SEOV_M
SEOV_S
GZRn107_Ch na_2019
GZRn133_Ch na_2019
GZRn127_Ch na_2019
GZRn134_Ch na_2019
GZRn95_Ch na_2019
GZRn96_Ch na_2019
GZRn92_Ch na_2019
GZRn98_Ch na_2019
GZRn100_Ch na_2019
GZRn73_Ch na_2019
GZRn110_Ch na_2019
GZRn148_Ch na_2019
GZRn36_Ch na_2018
GZRn54_Ch na_2018
GZRn53_Ch na_2018
GZRn44_Ch na_2018
GZRn42_Ch na_2018
GZRn51_Ch na_2018
GZRn41_Ch na_2018
GZ488_Ch na_2016
GZ473_Ch na_2016
MM23_Ch na_2017
HN4_Ch na_2017
GZRn76_Ch na_2019
GZRn83_Ch na_2019
GZRn104_Ch na_2019
HN1_Ch na_2017
GZ15_Ch na_2017
YY27_Ch na_2017
GZ45_Ch na_2014
GZ325_Ch na_2015
GAW50_Ch na_2021
GAW30_Ch na_2021
L99_UK_1997
SEOV_201700423_US_2017
SEOV_201700420_US_2017
SEOV_201701022_US_2017
SEOV_201700683_US_2017
SEOV_201701593_US_2017
SEOV_201701321_US_2017
SEOV_201802480_US_2018
SEOV_201701093_US_2017
SEOV_201701554_US_2017
Rn2147_Netherlands_2016
Rn2125_Netherlands_2016
Cherwell_UK_2013
CVR_2019_UK_2019
Humber2_UK_2012
Humber1_UK_2012
Rn84_Sweden_2013
NB2002_US_2002
IR162_UK_1984
IR33_UK_1984
IR473_UK_1984
IR461_UK_1984
LN03_Ch na_2020
LN05_Ch na_2020
LN02_Ch na_2020
LN04_Ch na_2020
LN06_Ch na_2020
FJ35_Ch na_2020
FJ36_Ch na_2020
ZT71_Ch na_2006
Z37_Ch na_2000
ZT10_Ch na_2006
Fj372_Ch na_2013
DPRK08_North_Korea_2011
LN01_Ch na_2020
LY0852_France_2011
DN2_Ch na_2014
Rn10_145_South_Korea_2010
Rn10_134_South_Korea_2010
Rn11_53_South_Korea_2011
Seoul_80_39_1990
Rn11_44_South_Korea_2011
Hu02_258_South_Korea_2002
Tchoup toulas_POR_US_1984
SR_11_Japan_1981
J angx X anj anRn_07_2011_Ch na_2011
J angx X nj anRn_09_2011_Ch na_2011
GAW50_China_2021
GAW30_China_2021
LYO852_France_2011
DN2_China_2014
GZRn107_Ch na_2019
GZRn133_Ch na_2019
GZRn127_Ch na_2019
GZRn134_Ch na_2019
GZRn95_Ch na_2019
GZRn96_Ch na_2019
GZRn98_Ch na_2019
GZRn36_Ch na_2018
GZRn92_Ch na_2019
GZRn100_Ch na_2019
GZRn73_Ch na_2019
GZRn110_Ch na_2019
GZRn148_Ch na_2019
GZRn54_Ch na_2018
GZRn53_Ch na_2018
GZRn44_Ch na_2018
GZRn42_Ch na_2018
GZRn51_Ch na_2018
GZRn41_Ch na_2018
GZ488_Ch na_2016
GZ473_Ch na_2016
HN4_Ch na_2017
MM23_Ch na_2017
GZRn76_Ch na_2019
GZRn83_Ch na_2019
GZRn104_Ch na_2019
GZ45_Ch na_2014
GZ325_Ch na_2015
HN1_Ch na_2017
GZ15_Ch na_2017
YY27_Ch na_2017
L99_UK_1997
LY0852_France_2011
DN2_Ch na_2014
SEOV_201700423_US_2017
SEOV_201700420_US_2017
SEOV_201701022_US_2017
SEOV_201700683_US_2017
SEOV_201701593_US_2017
SEOV_201701321_US_2017
SEOV_201802480_US_2018
SEOV_201701093_US_2017
SEOV_201701554_US_2017
Rn2147_Netherlands_2016
Rn2125_Netherlands_2016
Cherwell_UK_2013
CVR_2019_UK_2019
NB2002_US_2002
GAW50_Ch na_2021
GAW30_Ch na_2021
Humber2_UK_2012
Humber1_UK_2012
Rn84_Sweden_2013
IR162_UK_1984
IR33_UK_1984
IR473_UK_1984
IR461_UK_1984
LN03_Ch na_2020
LN02_Ch na_2020
LN04_Ch na_2020
LN05_Ch na_2020
LN06_Ch na_2020
FJ35_Ch na_2020
FJ36_Ch na_2020
ZT71_Ch na_2006
ZT10_Ch na_2006
Z37_Ch na_2000
Fj372_Ch na_2013
DPRK08_North_Korea_2011
LN01_Ch na_2020
J angx X anj anRn_07_2011_Ch na_2011
J angx X nj anRn_09_2011_Ch na_2011
Rn10_145_South_Korea_2010
Rn10_134_South_Korea_2010
Rn11_53_South_Korea_2011
Hu02_258_South_Korea_2002
Seoul_80_39_1990
Rn11_44_South_Korea_2011
Tchoup toulas_POR_US_1984
SR_11_Japan_1981

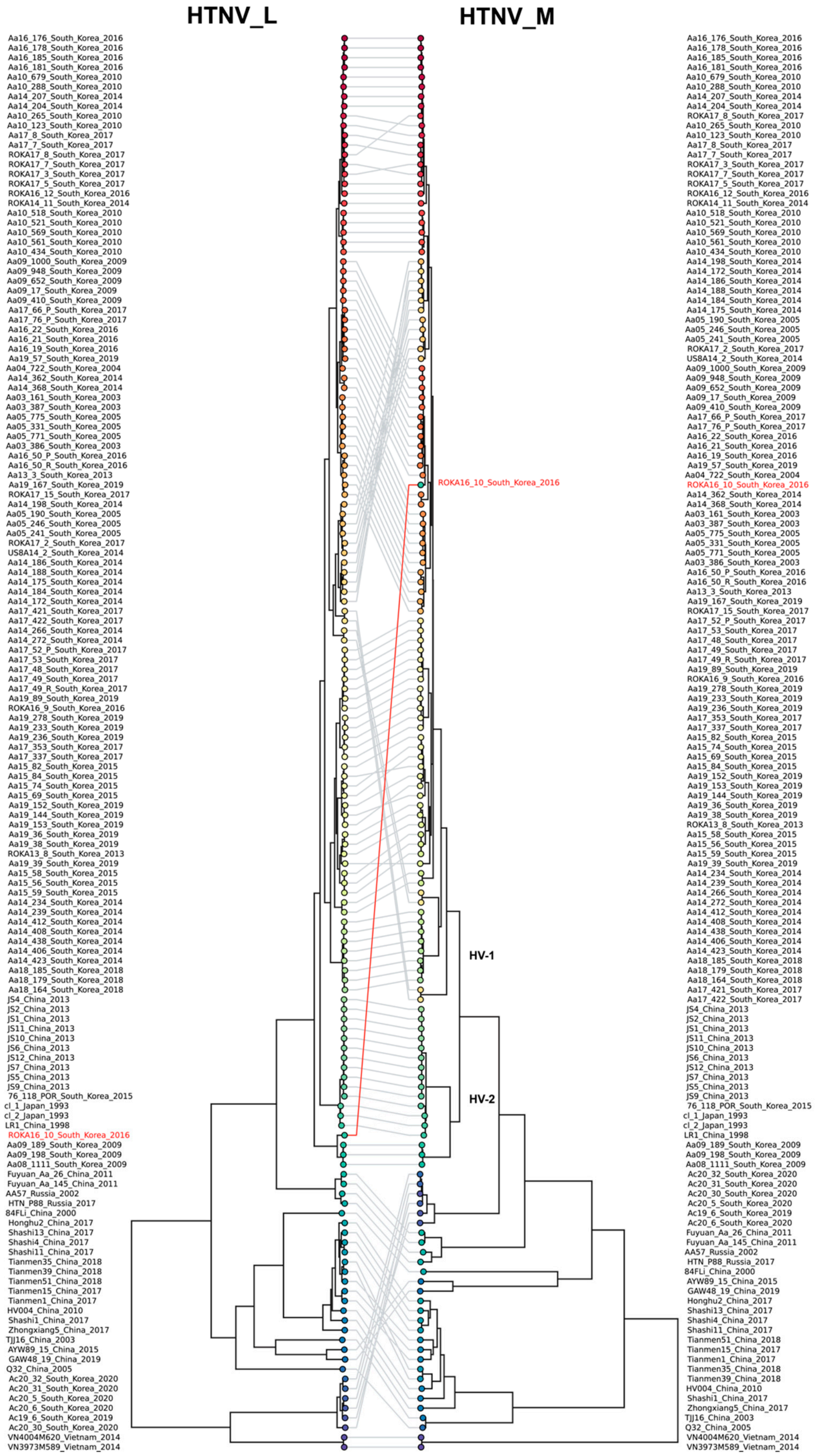
HTNV_L
HTNV_M
Aa16_176_South_Korea_2016
Aa16_178_South_Korea_2016
Aa16_185_South_Korea_2016
Aa16_181_South_Korea_2016
Aa10_679_South_Korea_2010
Aa10_288_South_Korea_2010
Aa14_207_South_Korea_2014
Aa14_204_South_Korea_2014
Aa10_265_South_Korea_2010
Aa10_123_South_Korea_2010
Aa17_8_South_Korea_2017
Aa17_7_South_Korea_2017
ROKA17_8_South_Korea_2017
ROKA17_7_South_Korea_2017
ROKA17_3_South_Korea_2017
ROKA17_5_South_Korea_2017
ROKA16_12_South_Korea_2016
ROKA14_11_South_Korea_2014
Aa10_518_South_Korea_2010
Aa10_521_South_Korea_2010
Aa10_569_South_Korea_2010
Aa10_561_South_Korea_2010
Aa10_434_South_Korea_2010
Aa09_1000_South_Korea_2009
Aa09_948_South_Korea_2009
Aa09_652_South_Korea_2009
Aa09_17_South_Korea_2009
Aa09_410_South_Korea_2009
Aa17_66_P_South_Korea_2017
Aa17_76_P_South_Korea_2017
Aa16_22_South_Korea_2016
Aa16_21_South_Korea_2016
Aa16_19_South_Korea_2016
Aa19_57_South_Korea_2019
Aa04_722_South_Korea_2004
Aa14_362_South_Korea_2014
Aa14_368_South_Korea_2014
Aa03_161_South_Korea_2003
Aa03_387_South_Korea_2003
Aa05_775_South_Korea_2005
Aa05_331_South_Korea_2005
Aa05_771_South_Korea_2005
Aa03_386_South_Korea_2003
Aa16_50_P_South_Korea_2016
Aa16_50_R_South_Korea_2016
Aa13_3_South_Korea_2013
Aa19_167_South_Korea_2019
ROKA17_15_South_Korea_2017
Aa14_198_South_Korea_2014
Aa05_190_South_Korea_2005
Aa05_246_South_Korea_2005
Aa05_241_South_Korea_2005
ROKA17_2_South_Korea_2017
US8A14_2_South_Korea_2014
Aa14_186_South_Korea_2014
Aa14_188_South_Korea_2014
Aa14_175_South_Korea_2014
Aa14_184_South_Korea_2014
Aa14_172_South_Korea_2014
Aa17_421_South_Korea_2017
Aa17_422_South_Korea_2017
Aa14_266_South_Korea_2014
Aa14_272_South_Korea_2014
Aa17_52_P_South_Korea_2017
Aa17_53_South_Korea_2017
Aa17_48_South_Korea_2017
Aa17_49_South_Korea_2017
Aa17_49_R_South_Korea_2017
Aa19_89_South_Korea_2019
ROKA16_9_South_Korea_2016
Aa19_278_South_Korea_2019
Aa19_233_South_Korea_2019
Aa19_236_South_Korea_2019
Aa17_353_South_Korea_2017
Aa17_337_South_Korea_2017
Aa15_82_South_Korea_2015
Aa15_84_South_Korea_2015
Aa15_74_South_Korea_2015
Aa15_69_South_Korea_2015
Aa19_152_South_Korea_2019
Aa19_144_South_Korea_2019
Aa19_153_South_Korea_2019
Aa19_36_South_Korea_2019
Aa19_38_South_Korea_2019
ROKA13_8_South_Korea_2013
Aa19_39_South_Korea_2019
Aa15_58_South_Korea_2015
Aa15_56_South_Korea_2015
Aa15_59_South_Korea_2015
Aa14_234_South_Korea_2014
Aa14_239_South_Korea_2014
Aa14_412_South_Korea_2014
Aa14_408_South_Korea_2014
Aa14_438_South_Korea_2014
Aa14_406_South_Korea_2014
Aa14_423_South_Korea_2014
Aa18_185_South_Korea_2018
Aa18_179_South_Korea_2018
Aa18_164_South_Korea_2018
JS4_China_2013
JS2_China_2013
JS1_China_2013
JS11_China_2013
JS10_China_2013
JS6_China_2013
JS12_China_2013
JS7_China_2013
JS5_China_2013
JS9_China_2013
76_118_POR_South_Korea_2015
cl_1_Japan_1993
cl_2_Japan_1993
LR1_China_1998
ROKA16_10_South_Korea_2016
Aa09_189_South_Korea_2009
Aa09_198_South_Korea_2009
Aa08_1111_South_Korea_2009
Fuyuan_Aa_26_China_2011
Fuyuan_Aa_145_China_2011
AA57_Russia_2002
HTN_P88_Russia_2017
84FLi_China_2000
Honghu2_China_2017
Shashi13_China_2017
Shashi4_China_2017
Shashi11_China_2017
Tianmen35_China_2018
Tianmen39_China_2018
Tianmen51_China_2018
Tianmen15_China_2017
Tianmen1_China_2017
HV004_China_2010
Shashi1_China_2017
Zhongxiang5_China_2017
TJJ16_China_2003
AYW89_15_China_2015
GAW48_19_China_2019
Q32_China_2005
Ac20_32_South_Korea_2020
Ac20_31_South_Korea_2020
Ac20_5_South_Korea_2020
Ac20_6_South_Korea_2020
Ac19_6_South_Korea_2019
Ac20_30_South_Korea_2020
VN4004M620_Vietnam_2014
VN3973M589_Vietnam_2014
ROKA16_10_South_Korea_2016
HV-1
HV-2
Aa16_176_South_Korea_2016
Aa16_178_South_Korea_2016
Aa16_185_South_Korea_2016
Aa16_181_South_Korea_2016
Aa10_679_South_Korea_2010
Aa10_288_South_Korea_2010
Aa14_207_South_Korea_2014
Aa14_204_South_Korea_2014
ROKA17_8_South_Korea_2017
Aa10_265_South_Korea_2010
Aa10_123_South_Korea_2010
Aa17_8_South_Korea_2017
Aa17_7_South_Korea_2017
ROKA17_3_South_Korea_2017
ROKA17_7_South_Korea_2017
ROKA17_5_South_Korea_2017
ROKA16_12_South_Korea_2016
ROKA14_11_South_Korea_2014
Aa10_518_South_Korea_2010
Aa10_521_South_Korea_2010
Aa10_569_South_Korea_2010
Aa10_561_South_Korea_2010
Aa10_434_South_Korea_2010
Aa14_198_South_Korea_2014
Aa14_172_South_Korea_2014
Aa14_186_South_Korea_2014
Aa14_188_South_Korea_2014
Aa14_184_South_Korea_2014
Aa14_175_South_Korea_2014
Aa05_190_South_Korea_2005
Aa05_246_South_Korea_2005
Aa05_241_South_Korea_2005
ROKA17_2_South_Korea_2017
US8A14_2_South_Korea_2014
Aa09_1000_South_Korea_2009
Aa09_948_South_Korea_2009
Aa09_652_South_Korea_2009
Aa09_17_South_Korea_2009
Aa09_410_South_Korea_2009
Aa17_66_P_South_Korea_2017
Aa17_76_P_South_Korea_2017
Aa16_22_South_Korea_2016
Aa16_21_South_Korea_2016
Aa16_19_South_Korea_2016
Aa19_57_South_Korea_2019
Aa04_722_South_Korea_2004
ROKA16_10_South_Korea_2016
Aa14_362_South_Korea_2014
Aa14_368_South_Korea_2014
Aa03_161_South_Korea_2003
Aa03_387_South_Korea_2003
Aa05_775_South_Korea_2005
Aa05_331_South_Korea_2005
Aa05_771_South_Korea_2005
Aa03_386_South_Korea_2003
Aa16_50_P_South_Korea_2016
Aa16_50_R_South_Korea_2016
Aa13_3_South_Korea_2013
Aa19_167_South_Korea_2019
ROKA17_15_South_Korea_2017
Aa17_52_P_South_Korea_2017
Aa17_53_South_Korea_2017
Aa17_48_South_Korea_2017
Aa17_49_South_Korea_2017
Aa17_49_R_South_Korea_2017
Aa19_89_South_Korea_2019
ROKA16_9_South_Korea_2016
Aa19_278_South_Korea_2019
Aa19_233_South_Korea_2019
Aa19_236_South_Korea_2019
Aa17_353_South_Korea_2017
Aa17_337_South_Korea_2017
Aa15_82_South_Korea_2015
Aa15_74_South_Korea_2015
Aa15_69_South_Korea_2015
Aa15_84_South_Korea_2015
Aa19_152_South_Korea_2019
Aa19_153_South_Korea_2019
Aa19_144_South_Korea_2019
Aa19_36_South_Korea_2019
Aa19_38_South_Korea_2019
ROKA13_8_South_Korea_2013
Aa15_58_South_Korea_2015
Aa15_56_South_Korea_2015
Aa15_59_South_Korea_2015
Aa19_39_South_Korea_2019
Aa14_234_South_Korea_2014
Aa14_239_South_Korea_2014
Aa14_266_South_Korea_2014
Aa14_272_South_Korea_2014
Aa14_412_South_Korea_2014
Aa14_408_South_Korea_2014
Aa14_438_South_Korea_2014
Aa14_406_South_Korea_2014
Aa14_423_South_Korea_2014
Aa18_185_South_Korea_2018
Aa18_179_South_Korea_2018
Aa18_164_South_Korea_2018
Aa17_421_South_Korea_2017
Aa17_422_South_Korea_2017
JS4_China_2013
JS2_China_2013
JS1_China_2013
JS11_China_2013
JS10_China_2013
JS6_China_2013
JS12_China_2013
JS7_China_2013
JS5_China_2013
JS9_China_2013
76_118_POR_South_Korea_2015
cl_1_Japan_1993
cl_2_Japan_1993
LR1_China_1998
Aa09_189_South_Korea_2009
Aa09_198_South_Korea_2009
Aa08_1111_South_Korea_2009
Ac20_32_South_Korea_2020
Ac20_31_South_Korea_2020
Ac20_30_South_Korea_2020
Ac20_5_South_Korea_2020
Ac19_6_South_Korea_2019
Ac20_6_South_Korea_2020
Fuyuan_Aa_26_China_2011
Fuyuan_Aa_145_China_2011
AA57_Russia_2002
HTN_P88_Russia_2017
84FLi_China_2000
AYW89_15_China_2015
GAW48_19_China_2019
Honghu2_China_2017
Shashi13_China_2017
Shashi4_China_2017
Shashi11_China_2017
Tianmen51_China_2018
Tianmen15_China_2017
Tianmen1_China_2017
Tianmen35_China_2018
Tianmen39_China_2018
HV004_China_2010
Shashi1_China_2017
Zhongxiang5_China_2017
TJJ16_China_2003
Q32_China_2005
VN4004M620_Vietnam_2014
VN3973M589_Vietnam_2014

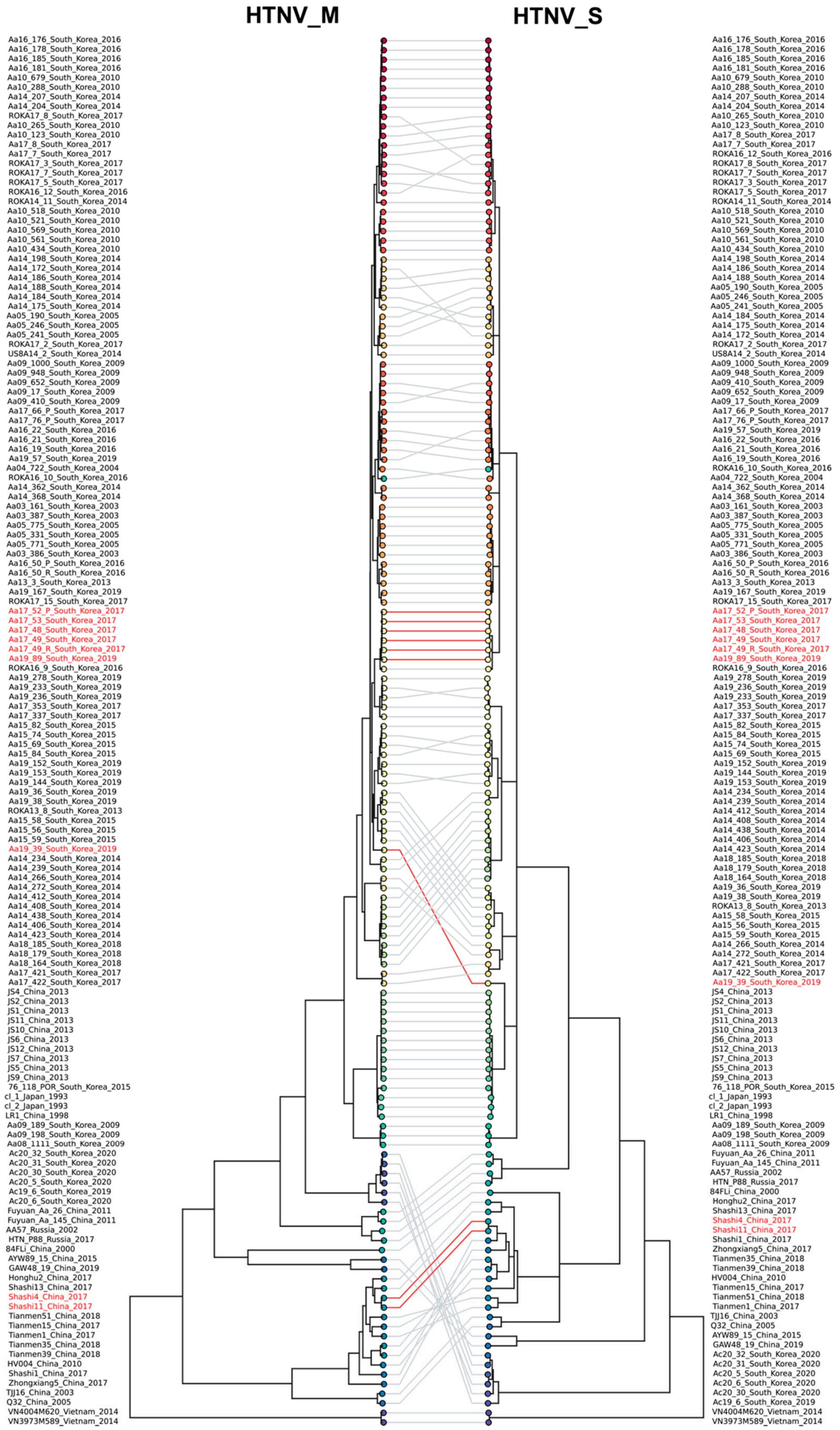
HTNV_M
HTNV_S
Aa16_176_South_Korea_2016
Aa16_178_South_Korea_2016
Aa16_185_South_Korea_2016
Aa16_181_South_Korea_2016
Aa10_679_South_Korea_2010
Aa10_288_South_Korea_2010
Aa14_207_South_Korea_2014
Aa14_204_South_Korea_2014
ROKA17_8_South_Korea_2017
Aa10_265_South_Korea_2010
Aa10_123_South_Korea_2010
Aa17_8_South_Korea_2017
Aa17_7_South_Korea_2017
ROKA17_3_South_Korea_2017
ROKA17_7_South_Korea_2017
ROKA17_5_South_Korea_2017
ROKA16_12_South_Korea_2016
ROKA14_11_South_Korea_2014
Aa10_518_South_Korea_2010
Aa10_521_South_Korea_2010
Aa10_569_South_Korea_2010
Aa10_561_South_Korea_2010
Aa10_434_South_Korea_2010
Aa14_198_South_Korea_2014
Aa14_172_South_Korea_2014
Aa14_186_South_Korea_2014
Aa14_188_South_Korea_2014
Aa14_184_South_Korea_2014
Aa14_175_South_Korea_2014
Aa05_190_South_Korea_2005
Aa05_246_South_Korea_2005
Aa05_241_South_Korea_2005
ROKA17_2_South_Korea_2017
US8A14_2_South_Korea_2014
Aa09_1000_South_Korea_2009
Aa09_948_South_Korea_2009
Aa09_652_South_Korea_2009
Aa09_17_South_Korea_2009
Aa09_410_South_Korea_2009
Aa17_66_P_South_Korea_2017
Aa17_76_P_South_Korea_2017
Aa16_22_South_Korea_2016
Aa16_21_South_Korea_2016
Aa16_19_South_Korea_2016
Aa19_57_South_Korea_2019
Aa04_722_South_Korea_2004
ROKA16_10_South_Korea_2016
Aa14_362_South_Korea_2014
Aa14_368_South_Korea_2014
Aa03_161_South_Korea_2003
Aa03_387_South_Korea_2003
Aa05_775_South_Korea_2005
Aa05_331_South_Korea_2005
Aa05_771_South_Korea_2005
Aa03_386_South_Korea_2003
Aa16_50_P_South_Korea_2016
Aa16_50_R_South_Korea_2016
Aa13_3_South_Korea_2013
Aa19_167_South_Korea_2019
ROKA17_15_South_Korea_2017
Aa17_52_P_South_Korea_2017
Aa17_53_South_Korea_2017
Aa17_48_South_Korea_2017
Aa17_49_South_Korea_2017
Aa17_49_R_South_Korea_2017
Aa19_89_South_Korea_2019
ROKA16_9_South_Korea_2016
Aa19_278_South_Korea_2019
Aa19_233_South_Korea_2019
Aa19_236_South_Korea_2019
Aa17_353_South_Korea_2017
Aa17_337_South_Korea_2017
Aa15_82_South_Korea_2015
Aa15_74_South_Korea_2015
Aa15_69_South_Korea_2015
Aa15_84_South_Korea_2015
Aa19_152_South_Korea_2019
Aa19_153_South_Korea_2019
Aa19_144_South_Korea_2019
Aa19_36_South_Korea_2019
Aa19_38_South_Korea_2019
ROKA13_8_South_Korea_2013
Aa15_58_South_Korea_2015
Aa15_56_South_Korea_2015
Aa15_59_South_Korea_2015
Aa19_39_South_Korea_2019
Aa14_234_South_Korea_2014
Aa14_239_South_Korea_2014
Aa14_266_South_Korea_2014
Aa14_272_South_Korea_2014
Aa14_412_South_Korea_2014
Aa14_408_South_Korea_2014
Aa14_438_South_Korea_2014
Aa14_406_South_Korea_2014
Aa14_423_South_Korea_2014
Aa18_185_South_Korea_2018
Aa18_179_South_Korea_2018
Aa18_164_South_Korea_2018
Aa17_421_South_Korea_2017
Aa17_422_South_Korea_2017
JS4_China_2013
JS2_China_2013
JS1_China_2013
JS11_China_2013
JS10_China_2013
JS6_China_2013
JS12_China_2013
JS7_China_2013
JS5_China_2013
JS9_China_2013
76_118_POR_South_Korea_2015
cl_1_Japan_1993
cl_2_Japan_1993
LR1_China_1998
Aa09_189_South_Korea_2009
Aa09_198_South_Korea_2009
Aa08_1111_South_Korea_2009
Ac20_32_South_Korea_2020
Ac20_31_South_Korea_2020
Ac20_30_South_Korea_2020
Ac20_5_South_Korea_2020
Ac19_6_South_Korea_2019
Ac20_6_South_Korea_2020
Fuyuan_Aa_26_China_2011
Fuyuan_Aa_145_China_2011
AA57_Russia_2002
HTN_P88_Russia_2017
84FLi_China_2000
AYW89_15_China_2015
GAW48_19_China_2019
Honghu2_China_2017
Shashi13_China_2017
Shashi4_China_2017
Shashi11_China_2017
Tianmen51_China_2018
Tianmen15_China_2017
Tianmen1_China_2017
Tianmen35_China_2018
Tianmen39_China_2018
HV004_China_2010
Shashi1_China_2017
Zhongxiang5_China_2017
TJJ16_China_2003
Q32_China_2005
VN4004M620_Vietnam_2014
VN3973M589_Vietnam_2014
Aa16_176_South_Korea_2016
Aa16_178_South_Korea_2016
Aa16_185_South_Korea_2016
Aa16_181_South_Korea_2016
Aa10_679_South_Korea_2010
Aa10_288_South_Korea_2010
Aa14_207_South_Korea_2014
Aa14_204_South_Korea_2014
Aa10_265_South_Korea_2010
Aa10_123_South_Korea_2010
Aa17_8_South_Korea_2017
Aa17_7_South_Korea_2017
ROKA16_12_South_Korea_2016
ROKA17_8_South_Korea_2017
ROKA17_7_South_Korea_2017
ROKA17_3_South_Korea_2017
ROKA17_5_South_Korea_2017
ROKA14_11_South_Korea_2014
Aa10_518_South_Korea_2010
Aa10_521_South_Korea_2010
Aa10_569_South_Korea_2010
Aa10_561_South_Korea_2010
Aa10_434_South_Korea_2010
Aa14_198_South_Korea_2014
Aa14_186_South_Korea_2014
Aa14_188_South_Korea_2014
Aa05_190_South_Korea_2005
Aa05_246_South_Korea_2005
Aa05_241_South_Korea_2005
Aa14_184_South_Korea_2014
Aa14_175_South_Korea_2014
Aa14_172_South_Korea_2014
ROKA17_2_South_Korea_2017
US8A14_2_South_Korea_2014
Aa09_1000_South_Korea_2009
Aa09_948_South_Korea_2009
Aa09_410_South_Korea_2009
Aa09_652_South_Korea_2009
Aa09_17_South_Korea_2009
Aa17_66_P_South_Korea_2017
Aa17_76_P_South_Korea_2017
Aa19_57_South_Korea_2019
Aa16_22_South_Korea_2016
Aa16_21_South_Korea_2016
Aa16_19_South_Korea_2016
ROKA16_10_South_Korea_2016
Aa04_722_South_Korea_2004
Aa14_362_South_Korea_2014
Aa14_368_South_Korea_2014
Aa03_161_South_Korea_2003
Aa03_387_South_Korea_2003
Aa05_775_South_Korea_2005
Aa05_331_South_Korea_2005
Aa05_771_South_Korea_2005
Aa03_386_South_Korea_2003
Aa16_50_P_South_Korea_2016
Aa16_50_R_South_Korea_2016
Aa13_3_South_Korea_2013
Aa19_167_South_Korea_2019
ROKA17_15_South_Korea_2017
Aa17_52_P_South_Korea_2017
Aa17_53_South_Korea_2017
Aa17_48_South_Korea_2017
Aa17_49_South_Korea_2017
Aa17_49_R_South_Korea_2017
Aa19_89_South_Korea_2019
ROKA16_9_South_Korea_2016
Aa19_278_South_Korea_2019
Aa19_236_South_Korea_2019
Aa19_233_South_Korea_2019
Aa17_353_South_Korea_2017
Aa17_337_South_Korea_2017
Aa15_82_South_Korea_2015
Aa15_84_South_Korea_2015
Aa15_74_South_Korea_2015
Aa15_69_South_Korea_2015
Aa19_152_South_Korea_2019
Aa19_144_South_Korea_2019
Aa19_153_South_Korea_2019
Aa14_234_South_Korea_2014
Aa14_239_South_Korea_2014
Aa14_412_South_Korea_2014
Aa14_408_South_Korea_2014
Aa14_438_South_Korea_2014
Aa14_406_South_Korea_2014
Aa14_423_South_Korea_2014
Aa18_185_South_Korea_2018
Aa18_179_South_Korea_2018
Aa18_164_South_Korea_2018
Aa19_36_South_Korea_2019
Aa19_38_South_Korea_2019
ROKA13_8_South_Korea_2013
Aa15_58_South_Korea_2015
Aa15_56_South_Korea_2015
Aa15_59_South_Korea_2015
Aa14_266_South_Korea_2014
Aa14_272_South_Korea_2014
Aa17_421_South_Korea_2017
Aa17_422_South_Korea_2017
Aa19_39_South_Korea_2019
JS4_China_2013
JS2_China_2013
JS1_China_2013
JS11_China_2013
JS10_China_2013
JS6_China_2013
JS12_China_2013
JS7_China_2013
JS5_China_2013
JS9_China_2013
76_118_POR_South_Korea_2015
cl_1_Japan_1993
cl_2_Japan_1993
LR1_China_1998
Aa09_189_South_Korea_2009
Aa09_198_South_Korea_2009
Aa08_1111_South_Korea_2009
Fuyuan_Aa_26_China_2011
Fuyuan_Aa_145_China_2011
AA57_Russia_2002
HTN_P88_Russia_2017
84FLi_China_2000
Honghu2_China_2017
Shashi13_China_2017
Shashi4_China_2017
Shashi11_China_2017
Shashi1_China_2017
Zhongxiang5_China_2017
Tianmen35_China_2018
Tianmen39_China_2018
HV004_China_2010
Tianmen15_China_2017
Tianmen51_China_2018
Tianmen1_China_2017
TJJ16_China_2003
Q32_China_2005
AYW89_15_China_2015
GAW48_19_China_2019
Ac20_32_South_Korea_2020
Ac20_31_South_Korea_2020
Ac20_5_South_Korea_2020
Ac20_6_South_Korea_2020
Ac20_30_South_Korea_2020
Ac19_6_South_Korea_2019
VN4004M620_Vietnam_2014
VN3973M589_Vietnam_2014

Figure S3. Inferred reassortment events using GiRaF analysis for SEOV and HTNV. Tanglegrams depicting the datasets with SEOV and HTNV strains were generated using the Python-based tool called Baltic. Incongruences with statistical significance, as detailed in Tables S2 and S3 for both viruses, are highlighted with red connecting lines. Of note, the trees from two independent analysis of two gene segment trees in GiRaF method may produce slightly different tree topologies.

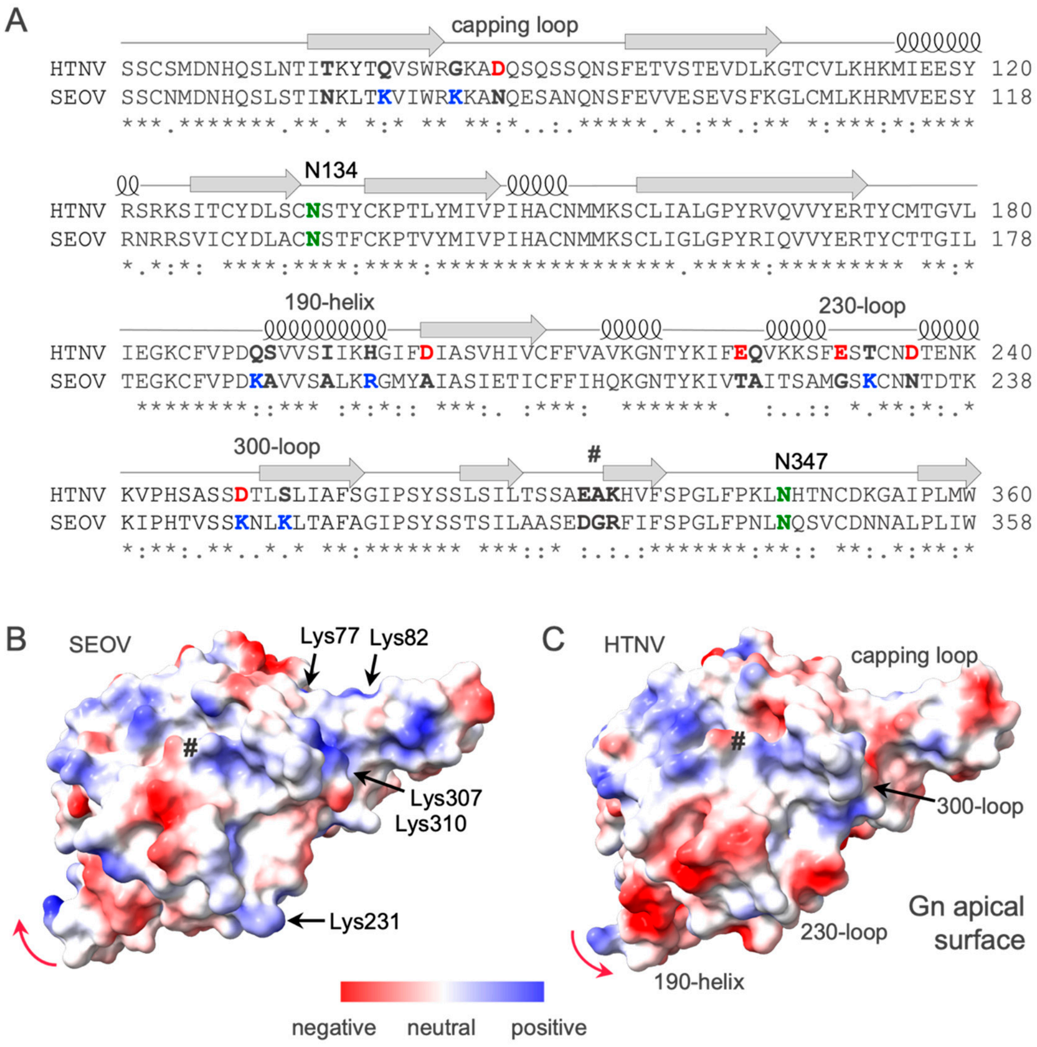

Figure S4. Structural properties of the SEOV & HTNV Gn ectodomains.

(A) Comparative analysis of consensus linear sequences for SEOV and HTNV Gn ectodomains. Putative N-linked glycosylation sites are denoted in green. Negatively charged amino acid residues are labeled in red, and positively charged residues are in blue. Key mutations in exposed motifs, denoted in (C) as the 'capping loop,' 230- and 300-loops, and 190-helix, are highlighted in bold in (A) and indicated with arrows in (B). Electrostatic surfaces of Gn domain tips are shown in (B) SEOV and (C) HTNV. The color coding in (B) and (C) represents the electric charge (red for negative, blue for positive, and white for neutral charge densities). The putative integrin binding motif is labeled with # in both (B) and (C).

Table S1. Isolation region, collection year and host information for SEOV and HTNV.

Virus	Isolation Region	Total	Collection Year						Host			
			~ 2000	2001–2005	2006–2010	2011-2015	2016-2020	2021 ~	Human	Rat	Mouse	Unknown
SEOV	China	48	1		2	6	37	2	0	44	1	3
	Europe	13	5			5	3		1	10		2
	North America	11	1	1			9			11		
	South/North Korea	7	1	1	2	3			1	5		1
	Japan	1	1							1		
	Total	80	9	2	4	14	49	2	2	71	1	6
HTNV	South Korea	110	1	10	17	30	52		12		68	30
	China	30	2	2	1	13	12		13	1	13	3
	Russia	2		1			1		1		1	
	Vietnam	2				2						2
	Japan	2	2									2
	Total	146	5	13	18	45	65	0	26	1	82	37

Rat, *Rattus norvegicus* or *Sapporo rat* (SEOV) and *Rattus confucianus* (HTNV)

Table S2. List of reassortment events in SEOV.

No.	Clade	Strain	L-M		L-S		M-S	
			GiRAF	CoalRe	GiRAF	CoalRe	GiRAF	CoalRe
1		GZ325/China/2015	Yes					
2		GZ45/China/2014	Yes					
3		GZ15/China/2017	Yes					
4		HN1/China/2017	Yes					
5		**YY27/China/2017**	Yes	Yes	Yes	Yes		
6		GZRn104/China/2019	Yes					
7		GZRn107/China/2019	Yes					
8	SV-1	GZRn127/China/2019	Yes					
9		GZRn133/China/2019	Yes					
10		GZRn134/China/2019	Yes					
11		GZRn76/China/2019	Yes					
12		GZRn83/China/2019	Yes					
13		GZRn95/China/2019	Yes					
14		GZRn96/China/2019	Yes					
15		GZRn36/China/2018					Yes	
16		Humber1/UK/2012			Yes			
17	SV-6	Humber2/UK/2012			Yes			
18		Rn84/Sweden/2013			Yes			
Total		22.50 % (18 out of 80 strains)	14		4		1	

Table S3. List of reassortment events in HTNV.

No.	Clade	Strain	L-M		L-S		M-S	
			GiRAF	CoalRe	GiRAF	CoalRe	GiRAF	CoalRe
1		ROKA17-8/South Korea/2017		Yes				Yes
2		Aa14-172/South Korea/2014		Yes				Yes
3		**ROKA16-10/South Korea/2016**	Yes	Yes		Yes		
4		Aa19-39/South Korea/2019					Yes	
5		Aa19-89/South Korea/2019					Yes	
6	HV-1	Aa15-82/South Korea/2015		Yes				Yes
7		Aa17-48/South Korea/2017					Yes	
8		Aa17-49-R/South Korea/2017					Yes	
9		Aa17-49/South Korea/2017					Yes	
10		Aa17-52-P/South Korea/2017					Yes	
11		Aa17-53/South Korea/2017					Yes	
12	HV-5	**Shashi11/China/2017**				Yes	Yes	Yes
13		**Shashi4/China/2017**				Yes	Yes	Yes
Total		8.90 % (13 out of 146 viral strains)	4		5		12	

Table S4. Comparative features of linear consensus sequences for the M glycoprotein precursors of SEOV and HTNV.

**Gene (region)**	**Sequence identity, %** (nucleotides)	**Sequence identity, %** (amino acids)	**Potential NLG sites***
Complete GPC	73.30	77.18	
Gn (20-490)	69.81	70.50	134, 347, 399
Gc (650-end)	76.65	82.61	928

* N-linked glycosylation numbering is based on the Asparagine (N) residue positions in HTNV M glycoprotein.

Table S5. Distinct amino acid residues in SEOV's Gn compared to HTNV.

No.	Amino acid position in SEOV Gn §	SEOV Gn (19-372)	HTNV Gn (21-374)	At the apical surface	Structural feature
1	26	Q	E		
2	39	S	I		
3	42	T	V		
4	47	L	M		
5	48	S	P		
6	50	Q	A		
7	52	A	T		
8	53	E	A		
9	55	L	M		
10	73	**N**	**T**		pocket
11	75	L	Y		
12	77	K	Q		
13	79	I	S		
14	82	K	G		
15	85	N	D	*	
16	87	E	S	*	capping loop
17	88	S	Q	*	
18	89	A	S	*	
19	90	N	S	*	
20	96	V	T	*	
21	98	E	S	*	
22	99	S	D		
23	102	F	L		
24	103	**L**	**T**		pocket
25	106	**M**	**V**		pocket
26	174	T	M		
27	179	T	I		
28	188	K	Q		
29	189	A	S		
30	193	A	I		
31	196	R	H		
32	198	M	I		
33	199	Y	F		
34	200	A	D		
35	212	**H**	**A**		pocket
36	213	**Q**	**V**		pocket
37	220	I	L	*	
38	221	V	F	*	
39	222	T	E	*	

40	223	A	Q	*	
41	225	T	K	*	230-loop
42	226	S	K	*	
43	227	A	S	*	
44	228	M	F	*	
45	229	G	E	*	
46	230	S	L	*	
47	231	K	T	*	
48	234	N	D	*	
49	256	A	V	*	
50	258	A	T		
51	264	A	S		
52	273	T	K	*	
53	283	P	A		
54	291	Q	S	*	
55	293	S	V	*	
56	295	Q	P	*	
57	296	I	A	*	
58	297	E	N	*	
59	304	V	A	*	300-loop
60	307	K	D	*	
61	310	K	S	*	
62	312	T	I	*	
63	327	A	T	*	
64	328	A	S	*	
65	330	E	T	*	
66	331	**D**	**E**	*	pocket
67	332	**G**	**A**	*	pocket
68	333	**R**	**K**	*	pocket
69	334	**F**	**H**		pocket
70	343	N	K	*	
71	346	Q	H	*	
72	348	V	T	*	
73	351	N	K	*	

§ The amino acid positions in HTNV's Gn differ from SEOV Gn by two, due to the inclusion of two additional amino acid residues at the beginning of the Gn N-terminus.

* Amino acid positions situated toward the apical surface of the Gn in SEOV Strain GZRn134/2019 (China) and HTNV Strain Aa19-233/2019 (South Korea).
